# Cross-species variability in lobular geometry and cytochrome P450 hepatic zonation: insights into CYP1A2, CYP2D6, CYP2E1 and CYP3A4

**DOI:** 10.3389/fphar.2024.1404938

**Published:** 2024-05-16

**Authors:** Mohamed Albadry, Jonas Küttner, Jan Grzegorzewski, Olaf Dirsch, Eva Kindler, Robert Klopfleisch, Vaclav Liska, Vladimira Moulisova, Sandra Nickel, Richard Palek, Jachym Rosendorf, Sylvia Saalfeld, Utz Settmacher, Hans-Michael Tautenhahn, Matthias König, Uta Dahmen

**Affiliations:** ^1^ Department of General, Visceral and Vascular Surgery, Experimental Transplantation Surgery, Jena University Hospital, Jena, Germany; ^2^ Department of Pathology, Faculty of Veterinary Medicine, Menoufia University, Shebin Elkom, Menoufia, Egypt; ^3^ Institute for Theoretical Biology, Institute für Biologie, Systems Medicine of the Liver, Humboldt-Universität zu Berlin, Berlin, Germany; ^4^ Institute for Pathology, BG Klinikum Unfallkrankenhaus Berlin, Berlin, Germany; ^5^ Department of General, Visceral and Vascular Surgery, Jena University Hospital, Jena, Germany; ^6^ Department of Veterinary Medicine, Institute of Veterinary Pathology, Freie Universität Berlin, Berlin, Germany; ^7^ Biomedical Center, Faculty of Medicine in Pilsen, Charles University, Pilsen, Czechia; ^8^ Department of Surgery, Faculty of Medicine in Pilsen, Charles University, Pilsen, Czechia; ^9^ Clinic and Polyclinic for Visceral, Transplantation, Thoracic and Vascular Surgery, Leipzig University Hospital, Leipzig, Germany; ^10^ Institute of Biomedical Engineering and Informatics, Ilmenau University of Technology, Ilmenau, Germany

**Keywords:** liver lobular geometry, metabolic zonation, drug metabolism, cytochrome P450, glutamine synthetase, interspecies, image analysis

## Abstract

There is a lack of systematic research exploring cross-species variation in liver lobular geometry and zonation patterns of critical drug-metabolizing enzymes, a knowledge gap essential for translational studies. This study investigated the critical interplay between lobular geometry and key cytochrome P450 (CYP) zonation in four species: mouse, rat, pig, and human. We developed an automated pipeline based on whole slide images (WSI) of hematoxylin-eosin-stained liver sections and immunohistochemistry. This pipeline allows accurate quantification of both lobular geometry and zonation patterns of essential CYP proteins. Our analysis of CYP zonal expression shows that all CYP enzymes (besides CYP2D6 with panlobular expression) were observed in the pericentral region in all species, but with distinct differences. Comparison of normalized gradient intensity shows a high similarity between mice and humans, followed by rats. Specifically, CYP1A2 was expressed throughout the pericentral region in mice and humans, whereas it was restricted to a narrow pericentral rim in rats and showed a panlobular pattern in pigs. Similarly, CYP3A4 is present in the pericentral region, but its extent varies considerably in rats and appears panlobular in pigs. CYP2D6 zonal expression consistently shows a panlobular pattern in all species, although the intensity varies. CYP2E1 zonal expression covered the entire pericentral region with extension into the midzone in all four species, suggesting its potential for further cross-species analysis. Analysis of lobular geometry revealed an increase in lobular size with increasing species size, whereas lobular compactness was similar. Based on our results, zonated CYP expression in mice is most similar to humans. Therefore, mice appear to be the most appropriate species for drug metabolism studies unless larger species are required for other purposes, e.g., surgical reasons. CYP selection should be based on species, with CYP2E1 and CYP2D6 being the most preferable to compare four species. CYP1A2 could be considered as an additional CYP for rodent versus human comparisons, and CYP3A4 for mouse/human comparisons. In conclusion, our image analysis pipeline together with suggestions for species and CYP selection can serve to improve future cross-species and translational drug metabolism studies.

## 1 Introduction

The liver is the primary metabolic organ in all mammals. Despite similarities between commonly used experimental species and humans, substantial anatomical and functional differences may interfere with the translation of results. With respect to liver anatomy and physiology, the following differences are evident. Humans, pigs, and mice have gallbladders, but rats lack this organ. Pigs have interlobular septa, but humans, rats, and mice lack this structure. The livers of rats, mice, and pigs are have multiple lobes, but the human liver does not have distinct lobes (Kruepunga et al., 2019).

Much less is known about functional similarity at the next smaller spatial scale. The microarchitecture of the hepatic lobules is very similar in different mammalian species. However, the degree of similarity in lobular geometry and zonal protein expression, such as cytochrome P450 (CYP), is poorly described. However, this knowledge is of great importance for the extrapolation of results from animal drug metabolism studies to the clinical situation. For example, observations made in rat hepatotoxicity studies cannot necessarily be reliably extrapolated to humans (Jaeschke et al., 2014).

Hepatic zonation refers to the spatial arrangement of metabolic processes and functions within the liver lobule (Gebhardt, 1992; Ben-Moshe et al., 2019; Martini et al., 2023). In the context of liver physiology, zonation describes how different zones of the hepatic lobule specialize in specific functions to ensure that the liver can effectively perform its diverse roles in metabolism, detoxification, bile production, and other functions. Depending on their location within the lobule, hepatocytes have different metabolic roles. The lobule is traditionally divided into three zones: Zone 1 (periportal)—high oxygen and nutrient content, main site of oxidative processes such as gluconeogenesis and fatty acid oxidation; Zone 2 (midzonal)—mix of functions; Zone 3 (pericentral)—lower oxygen content, primary site for glycolysis, lipogenesis, and CYP-mediated drug detoxification (Kietzmann, 2017; Ben-Moshe et al., 2019; Manco and Itzkovitz, 2021). CYP isoforms involved in drug activation and steroid metabolism show a particularly pronounced zonation pattern, with high expression and selective induction in the pericentral region (Lindros, 1997).

Cytochromes P450 are key drug-metabolizing enzymes involved in the first phase of drug metabolism. They convert drugs into water-soluble products for easy elimination (Almazroo et al., 2017; Garza et al., 2022). CYPs include many isozymes in the smooth endoplasmic reticulum and mitochondria of hepatocytes, small intestinal epithelium, and proximal renal tubules (Almazroo et al., 2017). Based on the relative distribution of liver CYPs, CYP1A2, 2C8, 2C9, 2E1, and 3A4 are the most abundant, whereas 2A6, 2B6, 2C19, 2D6, and 3A5 are less expressed (Shimada et al., 1994; Paine et al., 2006; Zanger and Schwab, 2013).

Prior work, such as that of Hammer and others, has used targeted proteomics to obtain cross-species information regarding the abundance of CYP proteins. Their investigations mainly focused on comparing various experimental settings, both *in vivo* and *in vitro*, in mice, rats, humans, and undifferentiated HepaRG cells (Hammer et al., 2021). Based on their observations, they found comparable and abundant expression levels of the CYP2D and CYP2E1 proteins across mice, rats, and humans. At the same time, CYP3A expression was similar but relatively moderate in clinical liver biopsies, rats, and mice. Moreover, they demonstrated moderate expression of CYP3A in humans, rats, and mice. Hrycay and Bandiera also comprehensively reviewed the similarities and differences in expression levels between mouse-human CYP proteins (Hrycay and Bandiera, 2009). By analyzing a database of CYP gene expression, they concluded that the laboratory mice serve as an essential tool for understanding CYP-mediated activities. The findings suggest that using laboratory mice can provide valuable insights into the mechanisms of CYP-mediated reactions thereby supporting drug development and medical research. However, in both studies, the zonated distribution of CYP expression was not investigated.

A spatially resolved cross-species comparison of CYP expression based on histological data is not yet available. The zonated expression pattern and the extent of expression variability also needs to be investigated in different species. This is important since the variability directly influences the number of liver lobules to be examined.

Several methods have been explored to elucidate liver lobular geometries and their zonation patterns. For example, Voronoi theory was used to analyze liver lobular geometry in both normal porcine and human livers to gain insight into the organization of classical liver lobules (Lau et al., 2021). In another study, zonal image analysis via Voronoi tessellation was applied to discern the distribution of hypoxia markers in the hepatic lobule of steatotic livers in mice. In both cases, the central veins were manually annotated in images of stained porcine and human or mouse and human liver sections, including images of hematoxylin-eosin and immunohistochemical staining for glutamine synthetase, a functional marker of pericentrally located hepatocytes (Peleman et al., 2023). Recently, the tissue positioning system, a deep learning-based analysis pipeline, was introduced to spatially analyze and quantify zonation within the liver lobule using differentially expressed markers in a mouse model (Wang et al., 2023). However, this study focused on zonation analysis and did not consider lobular geometry.

Understanding hepatic lobular geometry across species remains elusive. Similarly, the zonation patterns of CYP enzymes across species need to be better defined, since the extent of their similarities or differences remains largely unknown. Furthermore, the issue of intrahepatic interlobular variability in zonal expression of CYP enzymes is underexplored despite its relevance. The issue of interlobular variability is particularly important in human studies, where liver tissue is often obtained from minimally invasive needle biopsies. Given the limited nature of such samples, it is imperative to determine the minimum number of lobules required for sound qualitative and quantitative diagnosis. A notable absence in the field is the lack of a readily applicable mathematical framework capable of discerning geometries and zonation patterns from whole slide images (WSI). This is necessary for standardized, accurate, and reproducible analysis.

In this study, we compared lobular geometry and CYP expression in four species. We present our workflow for automated analysis of lobular geometry and zonation patterns in WSI. Using this approach, we systematically compared lobular geometry and zonation patterns of major CYP450 enzymes in four species.

## 2 Materials and methods

### 2.1 Samples

Archived formalin-fixed and paraffin-embedded liver samples from normal mice, rats, pigs, and human liver samples (*n* = 6/species) were used. For mice, liver samples from left lateral lobe (LLL), median lobe (ML), right lobe (RL), and caudate lobe (CL) were used. For rats, liver samples from ML were used. For Pigs, liver samples from LLL were used.

All *in-vivo* rodent studies were carried out using laboratory male C57BL6/J mice (ex-breeder) (Janvier, France) with 28–30 g of body weight and aged 8–9 months, as well as laboratory male Lewis rats (Charles River, France) with a body weight between 300–400 g and aged 3 months (*n* = 6/group). The animals were randomly housed in groups of three and provided with free food and water access. The cages were maintained in a conventional animal facility under constant environmental conditions, including a 12-h light/dark cycle, a constant temperature of 21 ± 2°C, and a 45%–65% relative humidity. Animals were humanely euthanized by an overdose of isoflurane with exsanguination.

Liver tissue specimens were taken from three males and three females clinically healthy Prestice Black-Pied pigs, weighing 25–33 kg and aged 3 months (*n* = 6). Pigs were kept under constant environmental conditions with a 12-h light/dark cycle in a conventional animal facility, constant room temperature (21°C ± 3°C), and 60% relative humidity. General anesthesia was performed using 10 mg/kg of ketamine (Narkamon-Spofa, Prague, Czech Republic), 5 mg/kg azaperone (Strensil - Jannsen Pharmaceutica NV, Beerse, Belgium), and 1 mg of atropine (Atropine Biotika - Hoechst Biotika, Martin, Slovak Republic). General anesthesia was induced and maintained by intravenous injection of propofol (1% mixture 5–10 mg/kg Propofol, Fresenius Kabi Norges, Halden, Norway). Animals were sacrificed with intravenous administration of T61 solution (MSD Animal Health, Kenilworth, NJ, United States) under general anesthesia (Palek et al., 2020).

Human liver samples were collected during clinically indicated hepatic surgeries in 2019 at the University Hospital of Jena, Germany. The human samples consisted of six individuals, two males and four females, aged between 45 and 59 years. Most patients subjected to liver surgery do not show an unimpaired morphology and present with additional features such as steatosis, fibrosis, and/or hepatic inflammation. We carefully selected the samples from patients based on hepatic morphology and had to compromise on gender. However, we did not observe an influence of gender on the zonal expression of CYP enzymes as indicated by the similar median and similar distribution visualized in the boxplot in Supplementary Figures 1, 2. Samples without additional pathologies were selected by independent assessment of WSI of HE-stained sections by four scientists (U.D., M.A., H-M.T., and O.D.), one of whom was a board-certified pathologist. The absence of any abnormal findings was confirmed by a board-certified hepato-pathologist (OD). Absence of steatosis was confirmed when less 5% of hepatocytes containing lipid droplets according to the current regulations (Kleiner et al., 2005; European Association for the Study of the Liver, European Association for the Study of Diabetes, and Obesity, 2016). In addition, absence of fibrosis was stated when liver tissue samples were scored (0) for necrosis, inflammation, and fibrosis according to Ishak score (modified Knodell score) (Ishak et al., 1995) using Hematoxylin and Eosin (HE) and Elastin Van Gieson staining (EvG) stained sections (Supplementary Figure 3; Supplementary Table 1).

### 2.2 Ethics statement

All animal experiments and animal housing were performed in accordance with current German regulations and guidelines for animal welfare and the ARRIVE guidelines for reporting animal research. Approval for the mouse study was granted by the Thüringer Landesamt für Verbraucherschutz, Thuringia (Approval-Number: UKJ-19-020), see also (Albadry et al., 2022), for the rat study Reg.-Nr. 02-043-10.

The pig study was approved by the Commission of Work with Experimental Animals under the Czech Republic’s Ministry of Agriculture, project ID: MSMT-15629/2020-4.

The human study was approved by the institutional review board of the University Hospital of Jena, Germany (ethical vote: UKJ_2018-1246-Material). This certifies that the study was performed in accordance with the ethical standards as laid down in the 1964 Declaration of Helsinki and its later amendments or comparable ethical standards.

### 2.3 Histology

Paraffin-embedded liver tissue samples were subjected to HE-staining after cutting 3 µm sections to assess lobular geometry. HE-staining was initiated by deparaffinization and rehydration using descendant grades of alcohol. Sections were immersed in hematoxylin and eosin solution, followed by dehydration using ascendant grades of alcohol and cleared with xylene.

Paraffin-embedded human liver tissue samples were also subjected to EvG-staining after cutting 3 µm sections to ensure the absence of fibrous connective tissue. First, liver tissue sections were deparaffinized and hydrated with distilled water. Sections were immersed in Verhoeff’s solution for 1 h until the tissue was completely black. Afterward, the sections were immersed in tap water and differentiated using 2% ferric chloride for 1–2 min. Slides were then washed with tap water and treated with 5% sodium thiosulfate for 1 min. Following washing with tap water, the sections were counterstained in Van Giseon’s solution for 3–5 min. The sections were then dehydrated using ascending grades of alcohol, cleared with xylene.

Immunohistochemistry was used to evaluate and quantify the spatial distribution of glutamine synthetase (GS) and four different cytochrome P450 (CYP) enzymes, as previously described (Albadry et al., 2022).

The staining procedure was performed on a series of consecutive 3 µm thick sections of formalin-fixed, paraffin-embedded liver tissue. Five different antibodies were used to visualize GS, CYP1A2, CYP2D6, CYP2E1 and CYP3A4, see Supplementary Table 2. Following the process of deparaffinization and rehydration using descending grades of ethanol, heat-based epitope retrieval was initiated. This involved the use of trisodium citrate buffer at pH 6.1 and a steamer set at 100°C for 30 min. The samples were then allowed to cool at room temperature for 20 min. The activity of endogenous tissue peroxidase was inhibited by treatment with 3% hydrogen peroxide solution. Non-specific binding to hydrophobic protein side chains or Fc receptors in the tissue was effectively inhibited using a commercially available protein block (ab64226, Abcam, Germany). The next step was an overnight incubation at 4°C using the appropriate CYP antibody as listed in Supplementary Table 2. For rabbit polyclonal primary antibodies (CYP2D6, 2E1, and 3A4), the rabbit-specific HRP/DAB IHC detection system (ab236469, Abcam) was used for 40 min at room temperature. For mouse monoclonal primary antibodies (GS and CYP1A2), additional primary antibody biotinylation was performed using Dako Animal Research Kit Peroxidase (K3954, Dako, Denmark).

In addition, endogenous avidin and biotin activity was inhibited using the avidin/biotin blocking kit (ab64212, Abcam), followed by application of the avidin-HRP complex. DAB chromogen (GV825, Dako, Denmark) was applied for approximately 3 min to visualize the reaction. Counterstaining was performed with Dako hematoxylin (CS700, Dako, Denmark) for 6–8 min. For each staining experiment, a single slide was designated as the negative reagent control condition in which the primary antibody was intentionally omitted. A uniform protocol was adopted for the staining process of the liver tissue samples in the four species.

The stained slides were mounted and digitized into a digital format using a whole slide scanner (L11600, Hamamatsu, Japan) equipped with NDP.view2 Plus Image viewing software (version U12388-02) at ×40 magnification.

#### 2.3.1 Qualitative assessment of zonated CYP expression

CYP expression was described qualitatively by taking the extent and zonal distribution (periportal, midzonal, and pericentral) and the signal intensity (mild, moderate, or strong) into account.

#### 2.3.2 Image analysis-based quantification of the extent of CYP zonal expression

The extent of CYP zonal expression was determined as the relative area covered by the specific staining signal. Quantification was performed as previously reported (Albadry et al., 2022), using a pattern recognition algorithm (generic classification 128) in the proprietary Histokat program developed by Fraunhofer MEVIS. In this approach, the entire slide scan is segmented into discrete square tiles of a predetermined size. The software was trained to recognize certain features or patterns using a minimum of 30 tiles per image from different liver lobes within a single section. This training involved the use of several representative images from the series.

#### 2.3.3 Statistical analysis

Descriptive ordinary one-way analysis of variance (ANOVA) was performed to compare the level of CYP expression among the four species (mice, rats, pigs, and humans). Tukey’s multiple comparison test was performed using GraphPad Prism version 9.3.1(471) for Windows, a software developed by GraphPad Software, San Diego, California, United States, www.graphpad.com. Data are presented as mean ± standard deviation. Differences were considered statistically significant when *p*-values were less than 0.05.

Geometrical parameters were tested for significance using the Kruskal–Wallis test and Mann–Whitney *U* test with significance levels as follows *p < 0.05, **p < 0.01, ***p < 0.001, ****p < 0.0001.

### 2.4 Pipeline for automatic quantification of lobular geometries and zonation patterns in WSI

**FIGURE 1:**
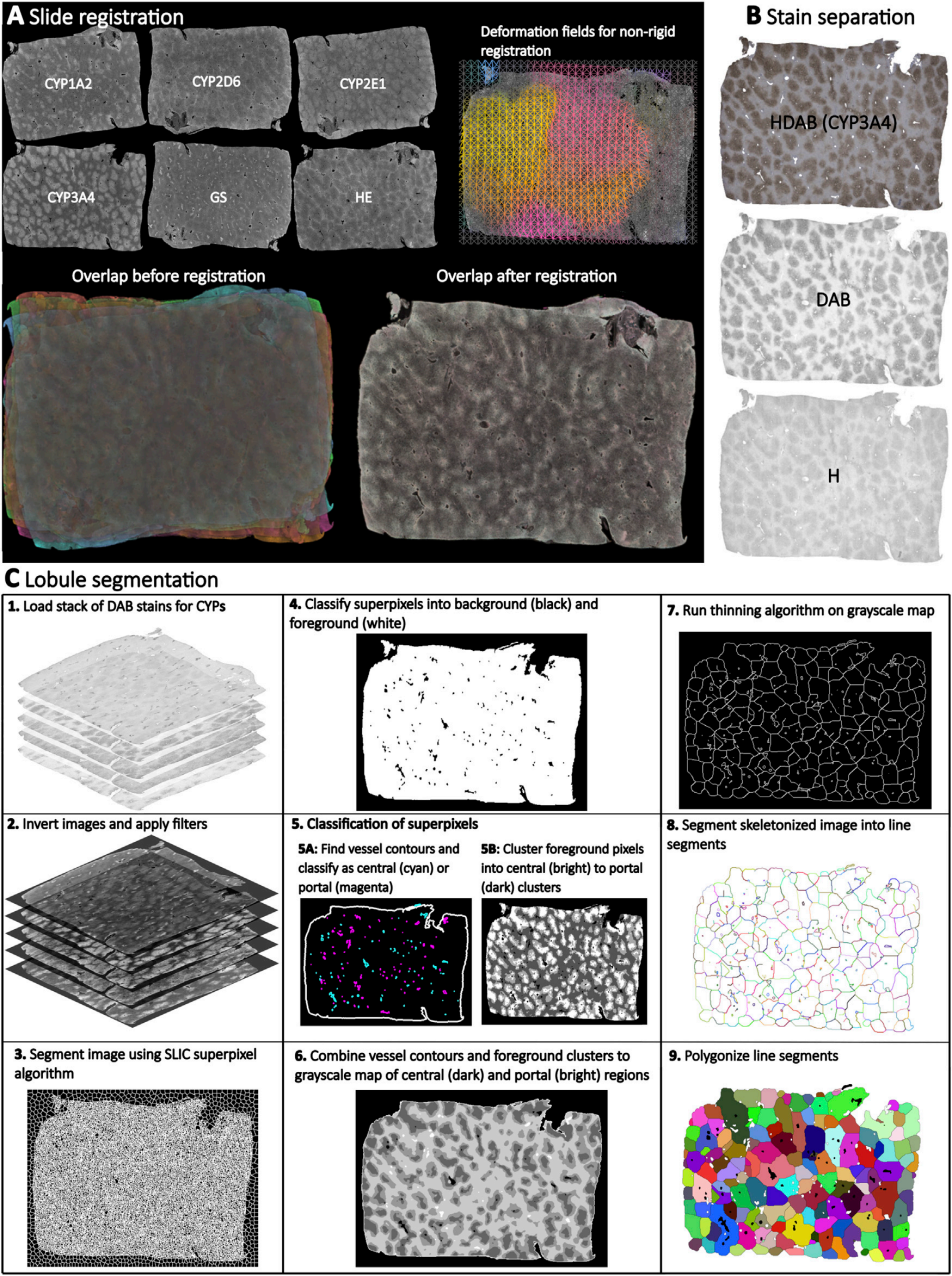
Overview of the analysis pipeline for quantifying lobular geometry and zonation patterns. The pipeline consists of the following steps: **(A)** Registration of HE, GS, and CYP whole slide images (WSI) using Valis, which allows the generation of multiplexed WSIs. **(B)** Color channel separation in the WSI. HE WSIs are separated into blue (hematoxylin) and pink (eosin), and IHC WSIs are separated into blue (hematoxylin) and brown (DAB) using color deconvolution. **(C)** Lobular segmentation of WSIs consisting of several steps: 1. Loading a stack of DAB stains for CYPs and GS. 2. Black and white image inversion and filter application. 3. Image segmentation using SLIC (Simple Linear Iterative Clustering) superpixel algorithm to generate uniform size and regular contour superpixels. 4. Classify superpixels into background (black color) and foreground (white color). 5. Classify the superpixels: 5.A. Find vessel contours and classify vessels as central (cyan) and portal (magenta), 5.B. Cluster foreground pixels, intro central (bright) to portal (dark) clusters. 6. Combine vessel contours and foreground clusters to create a grayscale map of central (dark) and portal (light) regions. 7. Apply a thinning algorithm to the grayscale map to create a skeleton. 8. Segment the skeletonized image into line segments. 9. Polygonize the line segments to create closed polygons.

We established an imgae analysis pipeline based on WSI of stained liver sections to quantify lobular geometry and zonation patterns of proteins (Figure 1). The pipeline consists of the following steps: (A) ROI detection in WSI. (B) Registration of adjacent WSI ROIs using Valis (Gatenbee et al., 2023), which allows the generation of multiplexed protein WSIs for the subsequent analysis. (C) Color channel separation in the immunostained WSI, providing access to the respective protein data based on color deconvolution. (D) Lobular segmentation of WSIs resulting in lobule boundaries and positional information (portality). Importantly, the presented approach does not require manual annotation of central veins and has successfully been applied to the analysis of lobular geometries and zonated quantification of proteins in WSI with single or multiple liver lobes of mice, rats, pigs, and humans.

**FIGURE 2:**
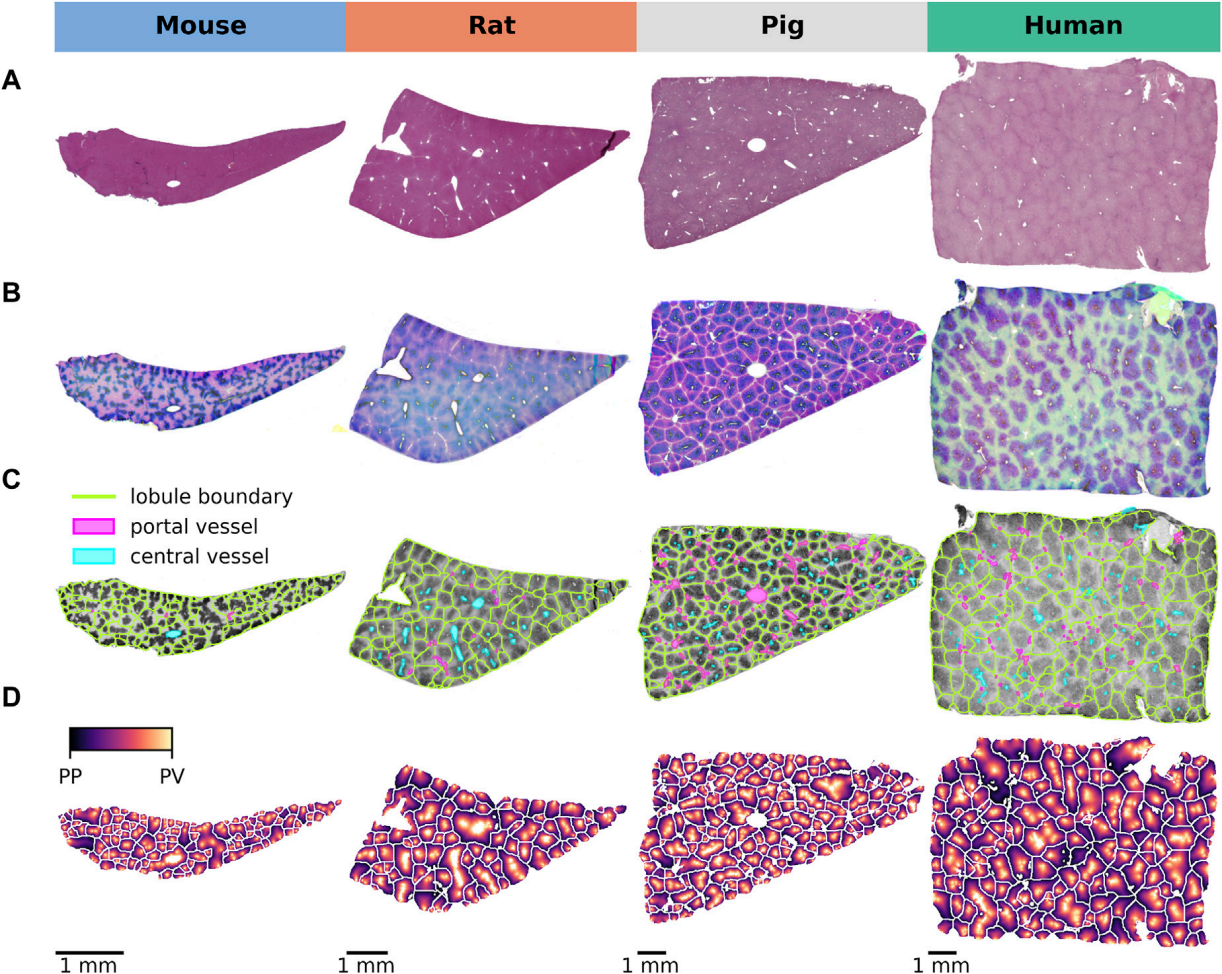
Lobular detection and position calculation. **(A)** HE staining of normal liver tissue from all four species showing lobular architecture. **(B)** Image normalization and stain separation to ensure optimal comparisons across species. **(C)** Detection of lobular regions providing a clear visual representation of lobular boundaries, distribution of lobules, central vessel and portal vessels on CYP2E1 staining. **(D)** Mapping of the central-portal distance on each lobule, allowing a quantitative analysis of the spatial arrangement of the lobules.

Using this approach, we systematically compared the lobular geometry and zonation patterns of key CYP450 enzymes across species (Figure 2). Lobular geometry was quantified in terms of lobular perimeter, area, compactness, and minimum bounding radius, followed by a statistical analysis of the differences between the species. Differences in zonation patterns in mice, rats, pigs, and humans were determined by analyzing the zonated expression of GS and the four CYPs (CYP1A2, 2D6, 2E1, and CYP3A4).

#### 2.4.1 ROI detection

The WSI images were provided as RGB images in NDPI format. For each subject, HE and hematoxylin 3,3′-Diaminobenzidine (HDAB) stained images were provided for the proteins GS, CYP1A2, CYP2D6, GYP2E1, and CYP3A4. All images from a single subject were from adjacent slides.

First, the source image files provided in NDPI format were annotated using QPath to mark the tissue samples on the slides. Subsequently, the whole slide images were loaded on the lowest resolution level with open slide (Goode et al., 2013), converted to grayscale, and thresholded using a binary threshold to get a foreground mask marking the tissue portion of the slide. Contour detection was utilized to transform the binary mask to polygons. The polygons were checked and selected if they contained the coordinates of the tissue annotation. The bounding boxes of the selected polygons were then used to load the region of interest (ROI) of the liver tissue of the source image in full resolution. The ROIs were saved as OME-TIFF. For murine subjects, four ROIs from different liver lobes were present on the slide, i.e., every ROI corresponded to one of the lobes. After ROI detection, the ROIs were matched based on the similarity of the tissue mask obtained by OTSU thresholding. In the case of very similar-shaped lobes, the matching failed, and the ROIS was manually mapped. For contour detection and thresholding, OpenCV (Bradski and Kaehler, 2008) implementations of the respective algorithms were used.

#### 2.4.2 Image registration

The set of images for each ROI was registered using VALIS, virtual alignment of pathology image series for multi-gigapixel whole slide images (Gatenbee et al., 2023), with default settings, and the registered ROIs were saved as OME-TIFF.

#### 2.4.3 Stain separation

Registration was followed by stain separation, resulting in two single-channel images of the H-stain and the DAB or E-stain, respectively. The resulting images were saved in the ZARR format with eight pyramidal layers. For stain separation, we used a color deconvolution algorithm introduced by Macenko (Macenko et al., 2009).

#### 2.4.4 Lobule segmentation

We used a classical image analysis approach to segment the lobule boundaries. The following procedure was applied to the image set for each subject and the ROI of the dataset. (1) The DAB stain for the image set was loaded for the proteins GS, CYP1A2, CYP2D6, GYP2E1, and CYP3A4 at resolution level 5 (×1.25 magnification). The registered protein ROIs were stacked into a 5-channel image. Protein images where the number of foreground pixels for a protein image was less than 80% of the median number of foreground pixels across all protein images were discarded. (2) Protein images were inverted so that bright regions correspond to high absorbance (high expression). Pixels where one of the channels was zero (i.e., background) were set to zero. Image filters were then applied to each channel. First, a median filter was applied, and the image was convolved to resolution level 6 (0.625×). Adaptive histogram normalization was used to reduce global differences in illumination and staining. Finally, after applying a median filter, the image was convolved to resolution level seven (0.3125×), and each channel was normalized to the maximum intensity of the channel. (3) The 5-channel images were segmented using OpenCV’s super-pixelization implementation, which assimilates similar pixels into a larger superpixel. (4) The superpixels were divided into foreground and background pixels. Superpixels were classified as background if more than 10% of the pixels in a superpixel were zero. (5) Foreground pixels were grouped into three zones: pericentral, midzone, and periportal.

Therefore, each superpixel was reduced into a 5-channel vector where each element represented the mean intensity of the channel in the superpixel. The vectors were clustered into three clusters using the K-means algorithm implementation of scikit-learn (Pedregosa et al., 2011). The cluster labels were sorted by the Euclidean distance of the cluster centers in ascending order. Given the high expression of the CYPs in the pericentral, and low expression in the periportal zone, the ordered labels corresponded to periportal, mid, and pericentral. The labels were mapped back to the superpixel representation, marking each foreground pixel as either pericentral, periportal, or midzone.

A mask was created from the background pixels, and contour detection was applied, yielding the contours of vessels and the tissue boundary. The contours were then classified into pericentral and periportal vessels. Each contour was transformed into a binary mask. Additionally a second mask was created by dilating this contour mask. Finally, the difference between contour and the dilated mask was calculated using the XOR operator. The difference mask was used to select the adjacent pixels of the vessel from the foreground image. The occurrence of each label (pericentral, midzone, periportal) was counted. It was expected that the number of pericentral labels would be higher for pericentral vessels and *vice versa* for periportal vessels. Based on this reasoning, the count vectors for all vessels were clustered into two clusters using the K-means algorithm.

(6) A grayscale representation was created from the clustered foreground and vessel contours, with pericentral colored black (zero) and and periportal vessels colored white (255), respectively. The foreground zones were uniformly distributed across the grayscale spectrum in between, with dark and light corresponding to pericentral and periportal zones, respectively. (7) An OpenCV implementation of a thinning algorithm was used to skeletonize the grayscale representation. The remaining lines are located in the expected center of the periportal zones and vessels (bright zones), marking the potential boundaries. (8) To polygonize the skeletonized image, we extracted line segments that we could polygonize. A line segment consists of adjacent pixels, where each pixel has, at max, two neighbors in the perpendicular or diagonally connected pixels. We implemented a pixel-walking algorithm that yields line segments, including the connecting pixels. The algorithm extends a line segment recursively. It analyzes the neighbors of the last appended pixel for a given line segment. If only one neighbor is found, it is appended to the segment, and the process is repeated. If no neighbor is found, the segment is terminated. The segment is also terminated if multiple neighbors are found, and new segments are created for each neighbor. When a segment is finished, the next initial segment is taken from the list of initial segments. The process is repeated until there are no initial segments left. (9) Finally, we used the shapely (Gillies et al., 2023) library to polygonize the set of line segments we obtained in the previous steps. All line segments that are not part of a closing circle are discarded.

#### 2.4.5 Generating the portality map

To analyze expression gradients, we calculated the relative position of each pixel in a lobule. The portality p was defined as

$$p\left(x,y\right)=1\;-\frac{dc\left(x,y\right)}{dc\left(x,y\right)+dp\left(x,y\right)}\in \left[0,1\right]$$

where dc and dp are the distance of a position to the nearest central and portal pixel, respectively. For each detected lobule, we generated a periportal and a pericentral boundary mask. In the pericentral boundary mask, all pixels were set false if they were either located in a central vessel or if the weighted intensity over all channels was in the 99% quantile. In the portal boundary mask, all pixels were set false if they were outside of the lobule boundary or if they were located in a portal vessel. The portal and central distance were calculated by applying the OpenCV distance transform function on these masks. This function calculates the distance for each pixel to the nearest background pixel. To generate the periportal and pericentral masks, we used the lobule boundary polygon and the vessel polygons. We then calculated the distance transformation for these masks using the OpenCV implementation.

For each protein, the intensity was background corrected and normalized by

$$I_{N}\left(x,y)=(I\left(x,y\right)\;-\;I_{bg}\right)/\left(I_{\max}-I_{bg}\right)\in \left[0,1\right]$$

Where I(x,y), I_bg_, and I_max_ denote the intensity of the pixel, the background intensity, and the maximum intensity of the slide, respectively, the maximum intensity was defined as the 99% quantile of the foreground pixels. The background intensity was estimated by the 20% percentile of the foreground pixels of the GS slide for each subject. GS expression covers only a very small area of the lobule, leaving the remainder as a robust background estimate. For each pixel, the normalized intensity and portality were written to a data frame for subsequent analysis.

#### 2.4.6 Lobular geometries

For every detected lobulus, the following geometric parameters were calculated based on the polygon for the lobulus boundary: perimeter, area, compactness, and minimum spanning distance.

### 2.5 Calculation of the number of required lobules

#### 2.5.1 Geometric parameters

The number of lobules (*n*) required for quantification of lobules geometries was calculated based on the method of determining sample size for estimating a population mean. The method depends on the margin of error ME (how accurate the results should be), a given confidence level of 95%, i.e., *α* = 0.05 (how confident the results need to be), and the estimate for the mean and standard deviation.

$$P\left(\left|\underline{x}-µ\right|>z_{\alpha /2}\cdot \sqrt{\frac{N-n}{N}\cdot \frac{\sigma^{2}}{n}}\right)=\alpha$$

$$z_{\alpha /2}\cdot \sqrt{\frac{N-n}{N}\cdot \frac{\sigma^{2}}{n}}=d=\mu \cdot ME$$

$$n=\frac{1}{\frac{d^{2}}{z_{\alpha /2}^{2}\cdot \sigma^{2}}+\frac{1}{N}}$$

For an analyzed subject and a geometric parameter, *N* is the total number of lobules, *μ* is the mean of the parameter over all lobuli of a subject, *σ* the corresponding standard deviation (provided in Supplementary Tables 3–5), *d* is the distance to the mean based on the margin of error ME, and *z* the *z* statistic for a given *α* value.

#### 2.5.2 Zonation patterns

The number of lobules (*n*) required for quantification of zonation patterns was calculated analogously to the geometric parameters. For each subject and each protein, one *n* was determined for each of the 12 zones. The mean *µ* is the mean of the intensity for the given position over the lobuli, with *σ* the corresponding standard deviation and *N* the number of lobuli for a subject. A margin of error ME of 10% and a confidence level of 95% (*α* = 0.05) were used for the calculation.

#### 2.5.3 Normalized intensity of CYP expression

Normalized expression was calculated for each lobule as the sum of all normalized staining intensities in the lobule for the respective protein. The mean *μ* and standard deviation *σ* of normalized expression for all lobules of a subject were used to analyze the required number of lobules *n*, where *N* is the number of all lobules for a subject. A margin of error ME of 20% and a confidence level of 95% (*α* = 0.05) were used for the calculation. In contrast to the Histokat surface fraction method, the normalized expression does not depend on a cutoff.

## 3 Results

Lobular geometry, and zonation of CYP enzyme expression are important features when assessing drug metabolism in different species. We used HE-stained samples to quantify lobular geometry in four species (mouse, rat, pig, and human). We then visualized five enzymes (GS, CYP1A2, CYP2D6, CYP2E1, and CYP3A4) in the four species. In this study, we focused on a systematic comparison of these features in the liver lobule using two different approaches.

First, WSIs were subjected to classical analysis, which involves a qualitative description of lobular geometry and staining patterns, followed by a statistical comparison of the relative surface area covered by the target protein. However, this approach is not ideal for describing differences in zonation patterns. In a second step, we developed an automated approach that focuses on the determination and comparison of lobule geometry in all four species, followed by zonation quantification and calculation of the minimum number of lobules required for the target protein.

### 3.1 Determination of species-specific differences in lobular geometry and extent of CYP enzymes expression using the classical descriptive approach

**FIGURE 3:**
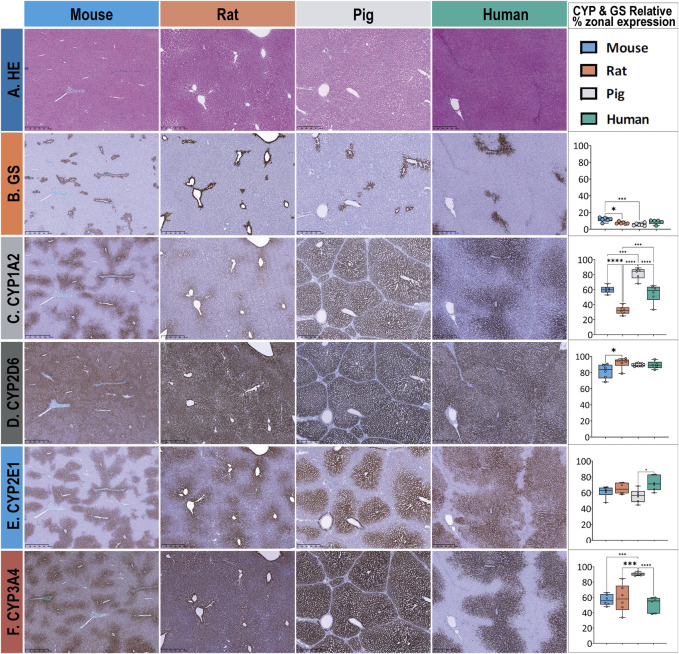
Overview of HE and extent of zonal expression as indicated by the relative surface covered by GS and CYP in liver tissue in mice, rats, pigs, and humans. The different stainings are depicted in rows, with columns 1–4 corresponding to the different species and column 5 presenting the results of the statistical analysis. **(A)** HE staining of normal liver tissue was used to depict lobular morphology in all species. Lobular structure appears similar except for the pig liver, where the lobules are separated by interlobular collagenous septae. **(B)** Glutamine synthetase (GS) staining is used to identify pericentral hepatocytes surrounding the central vein and to distinguish periportal from pericentral zones. Zonated expression is relatively similar across species, except for mouse and pig liver tissue, which show a significantly different distribution. **(C)** CYP1A2 staining shows a similar pericentral zonated expression in mice and humans while demonstrating a significantly different zonated expression pattern in rats (pericentral) and pigs (panlobular). **(D)** CYP2D6 exhibits almost panlobular and similar distribution across four species. **(E)** CYP2E1 is observed in the pericentral region in all four species, with no substantial difference. **(F)** CYP3A4 shows almost identical pericentral to midzonal expression patterns in mice and humans but panlobular in both rats and pigs. Scale bars 500 µm. Colors represent different species, with blue for mice, orange for rats, gray for pigs, and green for humans. Significance levels according to descriptive one-way ANOVA: * Significance level < 0.05, ** Significance level < 0.01, *** Significance level < 0.001, **** Significance level < 0.0001 (One-way-ANOVA).

Descriptive cross-species comparison of lobular geometry remains a challenge due to the subtle differences between the species. In contrast, the extent of zonated expression could be described easily and differed between the target proteins within a given species and the same protein between the different species, as shown in Figure 3.

In terms of lobular geometry, the most striking difference between species is the presence of interlobular septa in pigs. Porcine liver lobules have septa that encircle the hepatic lobule, allowing for easy identification of the lobule. Portal fields are connected by the septa, resulting in an appearance similar to bridging fibrosis in all other species. Portal fields, even of terminal vessels, contain relatively large amounts of connective tissue. In contrast, the portal field of rodents contain little amount of connective tissue and also few histiocytes. As a result the structural similarity between portal fields humans and rodents is higher compared to the pig. However, in humans, the hepatic artery is more prominent than in rats or pigs. Morphological analysis confirmed the striking similarity in lobular architecture between rodents.

In terms of qualitative and quantitative assessment of CYP expression (Figure 3; Supplementary Tables 6, 7), we confirmed that all CYP enzymes are expressed in the pericentral zone of the hepatic lobules in all four species but extend towards the periportal zone to varying degrees.

Zonation of GS expression (Figure 3, row B) follows a similar pattern in humans, pigs, rats, and mice. GS is expressed in the surrounding two to three layers of pericentral hepatocytes. Quantitative analysis of the relative area revealed a lobular coverage ranging from 5% to 13%. The maximum difference between species was approximately 2-fold, mouse versus pig. None of the species showed a significantly different coverage of GS-stained hepatocytes compared to human tissue.

CYP1A2 zonated expression (Figure 3, row C) is also most similar in humans and mice, with a strong pericentral signal extending from zone 3 into zone 2. In the rat, the signal was mostly confined to the pericentral three to four layers of hepatocytes in zone 3 and was of lower intensity. In pigs, the enzyme was expressed throughout the lobule and was not restricted to a specific zone. Quantitative analysis of the relative area covered by CYP1A2 showed a similar zonated expression pattern in humans and mice, with approximately 55.1 ± 11.9% and 60 ± 5.1% coverage of the hepatic lobule, respectively. In contrast, rats showed a significantly lower extent of expression, covering only 32.5 ± 5.5% of the lobular surface, whereas pigs showed a significantly higher extent of expression, covering approximately 81.76 ± 7.7% of the lobular surface.

CYP2D6 zonated expression (Figure 3, row D) shows a panlobular pattern in all four species with homogeneous expression in humans and pigs. In rats, the expression was rather inhomogeneous, whereas in mice, the signal was strong in the first pericentral layer of hepatocytes and rather weak in the remaining zones. Quantitative analysis of the relative lobular area covered by CYP2D6 showed a similar extent of coverage; for details, see Supplementary Tables 6, 7.

CYP2E1 zonated expression (see Figure 3, row E) follows a similar pattern and zonation across species, with a strong pericentral signal extending from zone 3 into zone 2. Quantitative analysis of the relative area covered by CYP2E1 revealed coverage of approximately 61.3 ± 7.2%, 65.4 ± 7%, and 56 ± 8.4% of the liver in mice, rats, and pigs, respectively, and approximately 72.2 ± 9.4% of the liver in humans.

CYP3A4 zonated expression (Figure 3, row F) follows a similar pattern and signal intensity in humans and mice, with a moderate to strong pericentral signal confined to zone 3 and extending into zone 2. In contrast, rat liver showed a strong signal restricted to the first line of pericentral hepatocytes, with a mild to moderate signal extending into hepatic zone 2. In pigs, CYP3A4 extended throughout the lobule, covering all three hepatic zones. Quantitative analysis of CYP3A4 showed a consistent pattern in humans, rats, and mice, covering approximately 50% of the lobular surface. Rat liver tissues showed higher interindividual variability in the extent of CYP3A4 zonal expression and distribution. In contrast, porcine liver showed a significantly higher extent of zonal expression, covering approximately 90.6 ± 2.0% of the lobular surface with CYP3A4-positive hepatocytes.

### 3.2 Species-specific lobular geometry

**FIGURE 4:**
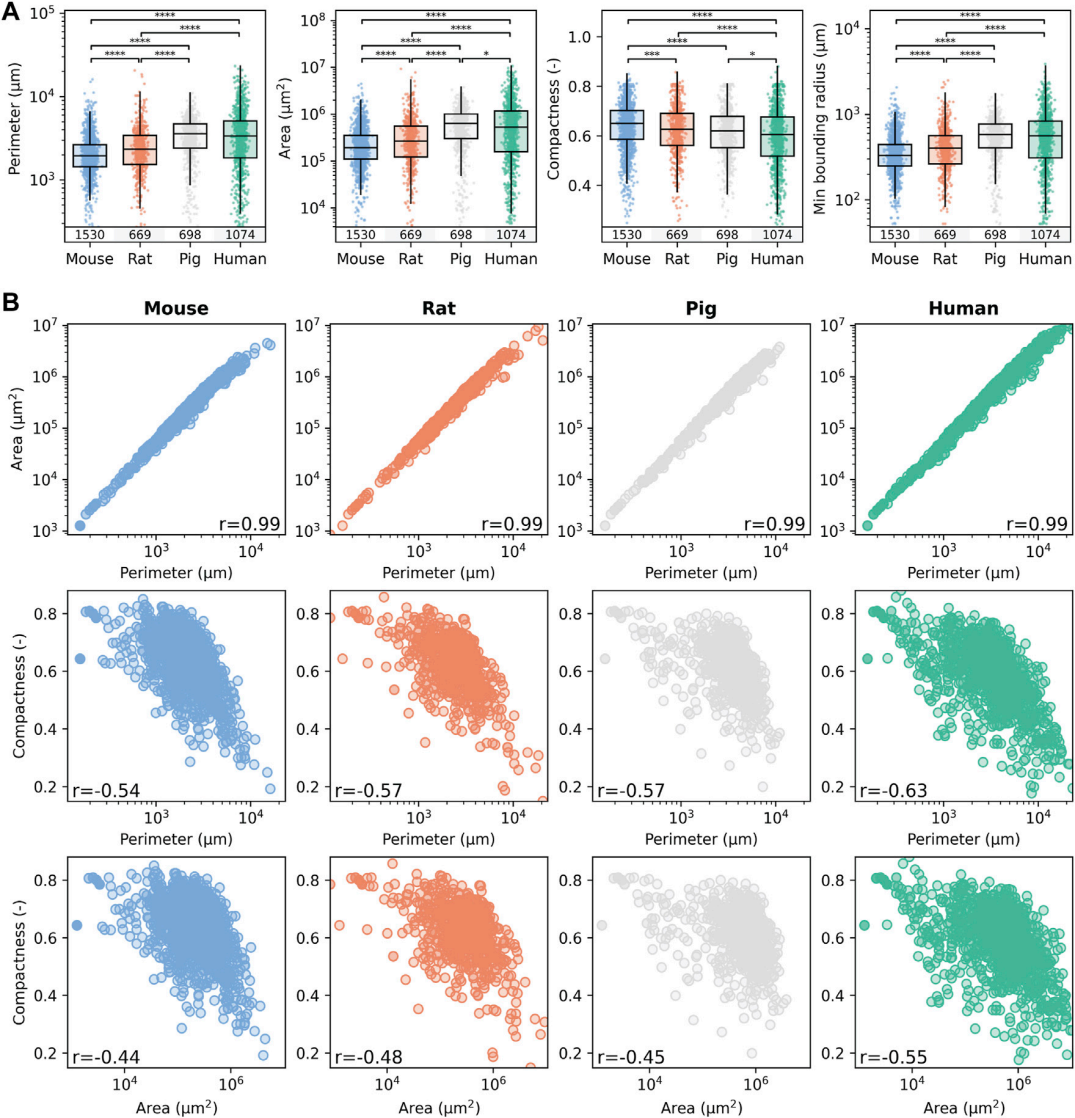
Comparison of lobular geometry in the four different species. **(A)** Quantification of species-specific lobular geometry. For all species, the lobular perimeter, area, compactness, and minimum bounding radius were calculated for all lobuli per section. Box blots and point clouds of these parameters are depicted. Boxes represent quartiles Q1 and Q3. The upper whisker and lower whisker extend to the last datum less than Q3 + 1.5 * IQR and the first datum greater than Q1 − 1.5 * IQR, respectively. IQR denotes interquartile range (Q3–Q1). Significance levels: *p < 0.05, **p < 0.01, ***p < 0.001, ****p < 0.0001. **(B)** Correlation between the geometric parameters was assessed using the Spearman rank correlation coefficient. Colors represent different species, with blue for mice, orange for rats, gray for pigs, and green for humans.

Based on the segmented lobule, species-specific lobular geometry was determined using the following geometric parameters: perimeter (the measurement of the circumferential length of the outer edge of the lobule), area (surface area of the lobule), compactness (the ratio of the area of the lobule polygon to the area of a circle with the same perimeter), and minimum bounding radius (radius of the minimum bounding circle enclosing the lobule). The resulting geometric parameters for the different species and the correlation between these parameters are shown in Figure 4, with numerical values given in Supplementary Tables 3–5.

Geometric parameters were calculated for 1,530 segmented lobules in mice, 669 in rats, 698 in pigs, and 1,074 in humans. Lobule size increases from mouse, rat, pig to human, i.e., the larger the species, the larger the lobule. Lobule radius and perimeter were the smallest in mice and almost twice as large in humans. For example, the mean ± standard deviation (sd) murine lobular boundary radius was 375 ± 216 μm, whereas the mean bounding radius for human lobules was 637 ± 473 µm. Differences in lobular geometry in humans could also be related to the actual site within the liver where the biopsy was taken.

Correspondingly, the mean perimeter of liver lobules increased with increasing species size, also by a factor of two when comparing mice (2,233 ± 1,397 µm) and humans (3,966 ± 3,200 µm). The minimum bounding radius of the lobules, a measure of their size, also nearly doubled from 375 ± 216 µm in mice, to 637 ± 473 µm in humans. As expected, the area of the lobules increased much more by a factor of 4 from 299,281 ± 36,009 μm^2^ in mice, 467,614 ± 729,369 μm^2^ in rats, 718,451 ± 57,536 μm^2^ in pigs, to 966,234 ± 1,357,480 μm^2^ in humans.

In contrast, the compactness of the lobules, which measures their roundness, decreased slightly with increasing species size. Compactness was highest in mice at 0.64 ± 0.10, followed by rats at 0.62 ± 0.11, pigs at 0.61 ± 0.10, and humans at 0.59 ± 0.12. These findings suggest that the lobules become slightly larger and less round with increasing species size.

Statistical comparison of the medians revealed statistically significant differences in lobular size, but little differences in compactness between species. A similar level of individual variability was observed in the geometric parameters in all species, with slightly larger variability in humans compared to the other species. The results of the geometric parameters from different subjects were very similar (Supplementary Table 4), i.e., no intra-individual variability in the geometric parameters could be observed. In addition sections from four different liver lobes were available for mice and were also compared (Supplementary Table 5). The determination of the geometric parameters for the different lobes resulted in comparable geometric parameters, i.e., no differences could be observed between LLL, ML, RL, and CL. I.e., neither intra-lobe variability in mice nor intra-subject variability in mice, rats, pigs and humans were observed in the geometric parameters, but significant differences were observed between species.

The correlation structure for the different geometric parameters was very similar between the different species (Figure 4B). Area and perimeter showed a very strong positive correlation (*r* = 0.99) in all species. As expected, hepatic lobules with a larger area also have a larger perimeter. In contrast, compactness showed a weak to moderate negative correlation to the perimeter [*r* in (−0.54, −0.63)] and area [*r* in (−0.44, −0.55)], i.e., the larger the lobule, the less compact they are possibly due to variable sectioning angles leading to a rather ovaloid appearance of the large lobule.

The similarities in the median and range of the geometric parameters across the species (Figure 4A) and the very similar correlation structure between species in the geometric parameters (Figure 4B) imply that the underlying complex 3D structure of the hepatic lobule must be rather similar. In conclusion, lobular geometry seems to be a robust feature, with low interindividual and interspecies variability but high variability between different lobules.

### 3.3 Quantification of zonated expression from WSIs: gradients and zonation patterns

**FIGURE 5:**
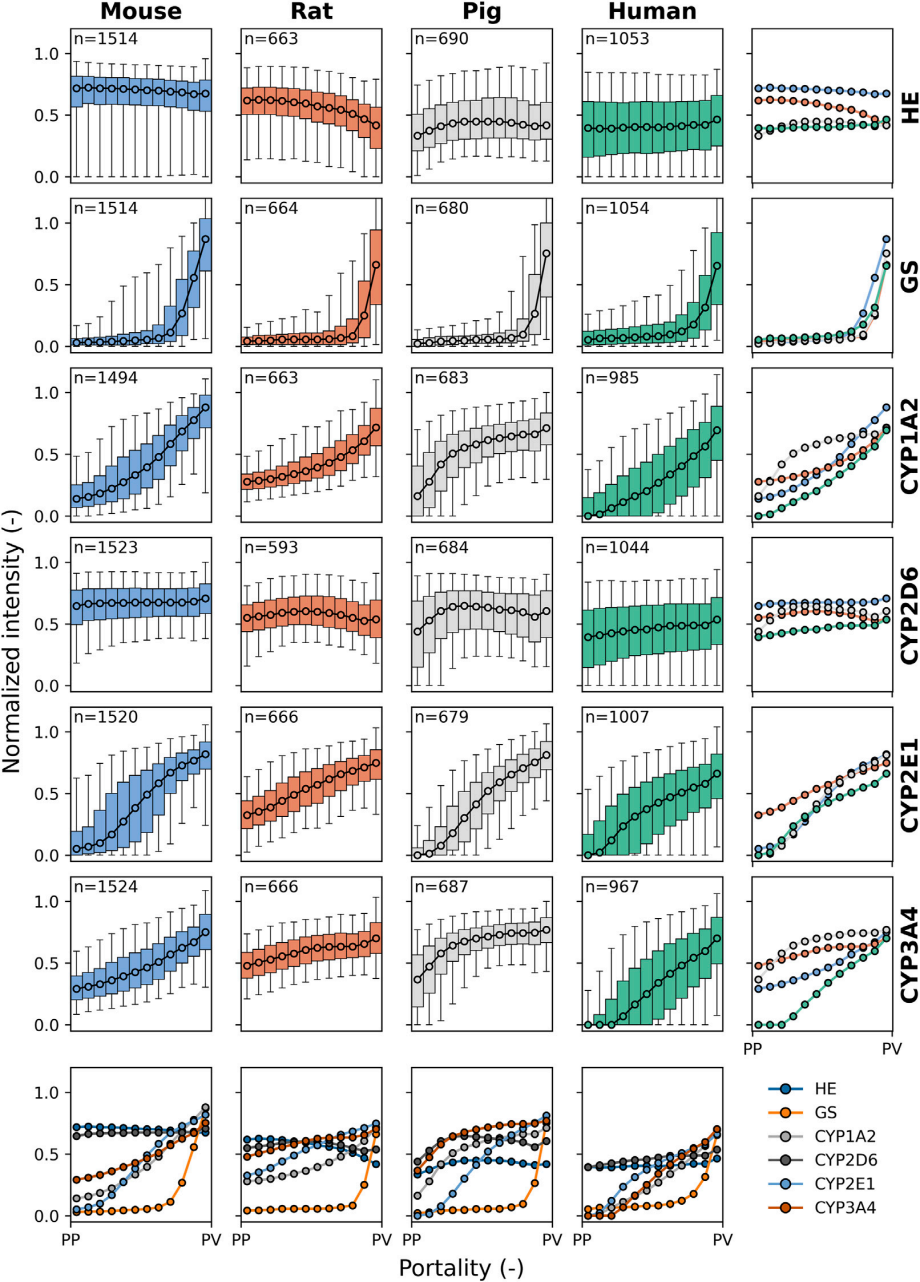
Species comparison of protein zonation. Zonation patterns of HE, GS, CYP1A2, CYP2D6, CYP2E1, and CYP3A4 in mouse (blue), rat (orange), pig (gray), and human (green). Normalized staining intensity (per slide) is plotted against portality (relative position between periportal (PP) and perivenous (PV) zones in each lobule). Data were binned in 12 bins from PP to PV. Median values are shown for all lobuli of all individuals. Box plots correspond to the median, interquartile range with whiskers at 5%, 95% percentile. Colors represent different species: blue for mice, orange for rats, gray for pigs, and green for humans. *n*: number of lobuli for the respective analysis.

The subsequent analysis quantified the zonated expression of CYP enzymes along the portal-venous axis in the entire liver lobules of the four species (Figure 5). Based on the calculated positions within the segmented lobules, we determined the position-dependent protein expression. Specifically, we assigned positions to all pixels within a lobule, ranging from periportal (0) to perivenous (1), based on their proximity to the nearest periportal or perivenous region. Using these positions, we determined the zonation patterns of GS and CYP proteins across lobules and species. Analysis of the combined zonated expression of all markers revealed distinct patterns for the different proteins and species.

HE-staining appeared as a consistent flat line across all species. This result was expected as HE-staining does not indicate protein expression along the sinusoid but rather delineates the morphological structure of the hepatic lobule, and no zonation differences are expected.

GS showed a similar gradient and zonation pattern along the entire portal-venous axis in the four species, as shown by the superimposed plots of normalized staining intensity. As described above, GS was static in zone 3, encompassing the two to three layers of pericentral hepatocytes, with no periportal distribution pattern in the four species.

CYP1A2 showed relatively similar gradient and zonation patterns in mice, rats, and humans, predominantly located in zone 3 and extending into zone 2 within the adjacent five to six rows of pericentral hepatocytes. In pigs, however, the gradient distribution of normalized intensity was mainly seen in zones 3 and 2 and extended into zone 1 of the periportal hepatocytes.

CYP2D6 showed a uniform and constant zonation pattern across the four species, with panlobular distribution within the liver lobules along the portal-venous axis. CYP2D6 was the only CYP analyzed that did not show a clear zonation pattern with higher protein content in the perivenous region compared to the periportal region.

CYP2E1 showed a linear gradient distribution of normalized intensity throughout the liver lobules in different species, predominantly in zones 3 and 2. In rats, there was a higher intensity in zone 1, with a flatter gradient than in the other species.

The intensity of the CYP3A4 gradient was similar in mice and humans, mainly in zone 3 and extending into zone 2. Conversely, rats and pigs exhibited a similar but distinct gradient distribution to mice and humans across the liver lobules, with CYP3A4 normalized intensity higher in zones 3 and 2 and extending to periportal hepatocytes in zone 1. The strongest periportal to perivenous gradient was observed in humans.

### 3.4 Number of lobules required to determine parameters

**FIGURE 6:**
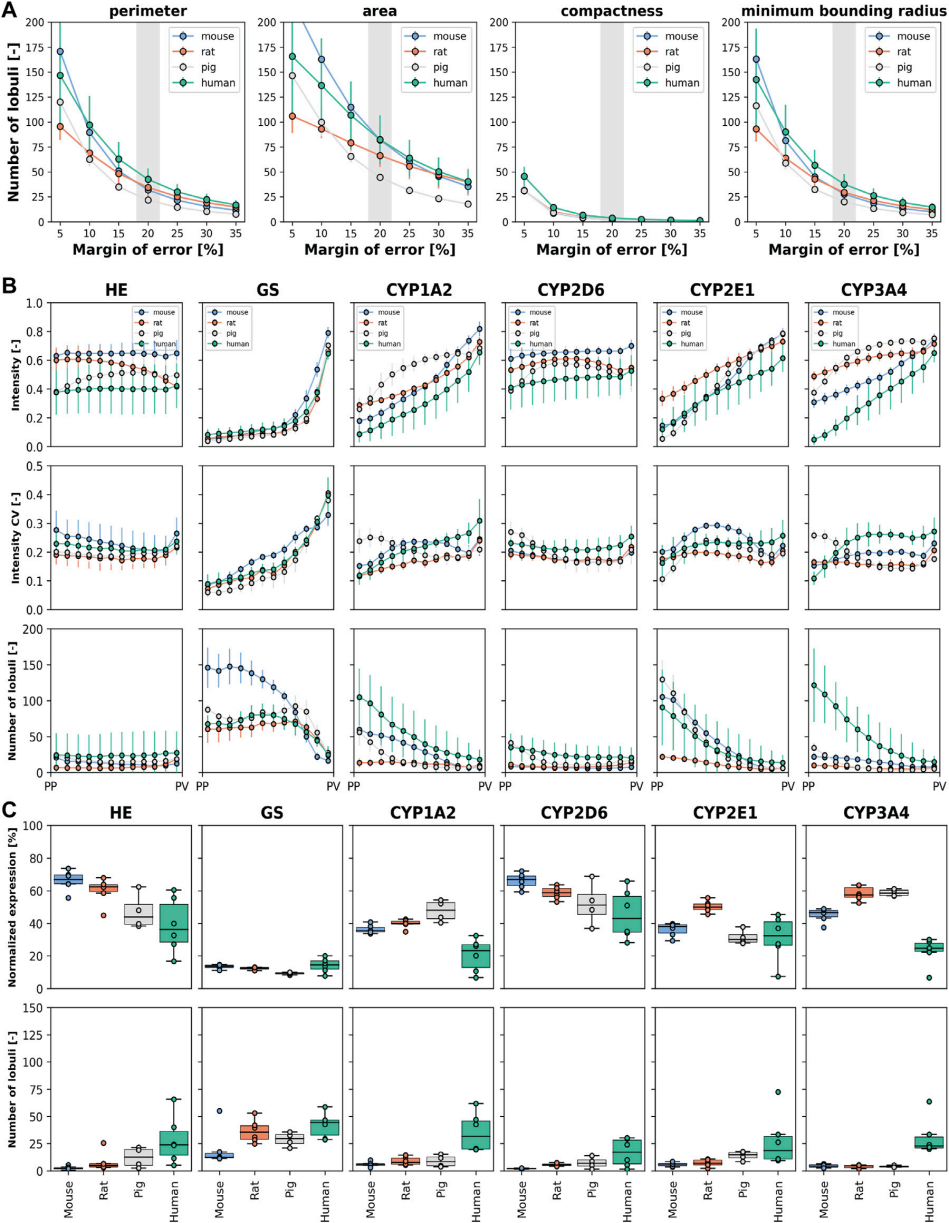
Number of lobules required to determine geometric parameters, zonation patterns, and relative expression. Species are color-coded as mouse (blue), rat (orange), pig (gray), and human (green). **(A)** Number of required lobules to determine the geometric parameters area, perimeter, compactness, and minimum boundary radius for a given margin of error (5%–35%) and a confidence level of 95%. Data expressed as mean ± SD for the subjects. **(B)** Number of required lobules to determine the protein zonation pattern, i.e., the normalized intensity for the 12 bins with a confidence level of 95% and a margin of error of 20%. Depicted are the normalized intensity mean ± SD for the subjects (top), the coefficient of variation of the normalized intensity as mean ± SD for the subjects (middle), and the required number of lobules for estimation of the zonation pattern as mean ± SD for the subjects (bottom). **(C)** Number of required lobules to determine the normalized protein expression per lobule with a confidence level of 95% and a margin of error of 20%. Normalized expression is depicted as mean values for individual subjects (averaged over all lobule per subject) and the corresponding boxplot of the means (top). Number of lobules required for individual subjects and the corresponding boxplot (bottom).

Next, we determined the number of lobules required for a representative analysis of geometric parameters, zonation patterns, and relative expression (Figure 6).

First, we determined the number of lobules required to calculate mean geometric parameters with 95% confidence at a given margin of error (Figure 6A, data in Supplementary Tables 4, 5). For example, to calculate compactness with 95% confidence and a 20% margin of error, the minimum number of liver lobules required are 2.2 ± 1.1 in mice, 2.7 ± 1.5 in rats, 2.4 ± 0.8 in pigs, and 3.8 ± 2.9 in humans.

Key findings included: (i) A larger margin of error reduces the number of lobules required for analysis. The dependence of the number of lobules required on the margin of error is highly non-linear, i.e., a small relaxation of the possible margin of error requires much fewer lobules. (ii) Human geometric parameters require a larger number of lobules for accurate determination compared to mice. The number of lobules required for rats and pigs is between those required for humans and mice. (iii) Different geometric parameters require different numbers of lobules for accurate evaluation. For example, area requires more lobules than perimeter and minimum boundary radius, while compactness requires the least number of lobules for accurate quantification. (iv) There is significant variability between biopsies of the same species.

We then determined the number of lobules required to calculate zonation patterns, also with 95% confidence and a 20% margin of error (Figure 6B). The number of lobules required for the zonation pattern varied with the protein of interest as well as the species. For many positions and proteins, less than 25 lobules were sufficient to determine the mean expression for a given zonal position. However, the analysis of GS and CYP2E1 in mice required a higher number of lobules, up to 150 for GS and 100 for CYP2E1, respectively. Similarly, in humans, quantification of CYP3A4 required up to 120 lobules for periportal protein levels. An important factor for the required number of lobules was the coefficient of variance of the intensity at the given position in combination with the intensity. A large variability in protein expression at a given position in combination with small intensities resulted in a much higher number of lobules for a reliable determination of the mean intensity. The protein pattern in a liver lobule is similar for most proteins, but with different degrees of interlobular and interindividual variability in the four species.

Finally, we determined the number of lobules required to determine the normalized expression of proteins within a lobule again with 95% confidence and 20% error (Figure 6C, data in Supplementary Table 8). Many normalized protein expressions could be determined with up to 10 lobules. Determination of the normalized expression of different CYP proteins in mice and rats was relatively similar and required fewer lobules than in other species.

An important finding is that significantly fewer lobules are required to determine the normalized expression of a protein in a lobule than to determine the lobular pattern. To determine normalized protein expression, the highest number of lobules was required for human liver tissue, followed by pig, rat, and mouse liver tissue.

## 4 Discussion

The primary aim of the current study was to investigate the critical interplay between lobular geometry and the zonation of major CYP proteins in four species, mice, rats, pigs, and humans.

Using WSI and our newly established image analysis pipeline, we were able to accurately quantify lobular geometry and zonated expression of CYP proteins in the liver across the four species, saving the step of manual annotation.

### 4.1 Automated image analysis pipeline

Our pipeline allowed a detailed, systematic comparison of lobular structures and the zonal distribution of key CYP450 enzymes in four species (mouse, rat, pig, and human). Importantly, our approach does not require time-consuming manual annotation of the WSI by experts in contrast to other published methods for hepatic lobule segmentation (Schwen et al., 2016; Lau et al., 2021; Peleman et al., 2023; Wang et al., 2023). This innovative approach to zonation assessment allows quantitative comparative analysis of the zonation patterns of multiple target proteins, such as the four different CYP proteins, in a single species, as well as the comparison of single proteins between different species. The superimposed curves clearly show that the zonated expression patterns in mice and humans are rather similar, while those in rats and pigs are more different. Our pipeline can be easily extended to quantify additional proteins and image modalities such as immunofluorescence as long as some stable markers of zonation, such as CYP1A2 and GS, are present in the staining data set. We expect that the pipeline could easily be adapted to other tissues such as the kidney.

Similar to our approach, the tissue positioning system (TPS) uses information from multiple stains to determine the position within the lobule based on K-means clustering (Wang et al., 2023). Our approach goes one step further and uses the position information to segment the lobular geometry. In contrast to TPS, our approach is based on a series of consecutive sections stained with different antibodies and registration of the corresponding WSI, whereas TPS requires a single fluorescence WSI with DAPI, GS and the proteins of interest. As mentioned above, our approach can be easily adapted to immunofluorescence images, whereas applying the TPS to conventional IHC might be more challenging. It would require the additional steps of image registration and stain separation, and the AI-based vessel detection would likely need to be recalibrated. We were able to demonstrate that our pipeline works successfully on samples from different species and liver lobes, whereas the TPS was only applied to mouse samples. It would be interesting to see if the TPS is transferable to other species, as the geometric differences in size could be a challenge for the trained image algorithm.

Similar to our approach, Hoehme et al. used information from multiple stains to determine the positions of the central and portal veins and calculated lobule sizes from these locations during regeneration (Hoehme et al., 2023). The study reported a lobular area of 0.21 ± 0.05 mm^2^ for sham-operated C56B6/N in young adult animals aged 11–14 weeks, compared to our mean lobular area of 0.30 ± 0.36 mm^2^ in a different substrain of C57Bl6/J mice of slightly higher age, but comparable to the values reported after regeneration of ∼0.30 mm^2^. A lobular area of 1.66 ± 0.84 mm^2^ was reported in female German Landrace pigs at 2–3 weeks of age, compared to our area of 0.72 ± 0.57 mm^2^ in Prestice Black-Pied pigs weighing 25–33 kg and aged 3 months.

### 4.2 Lobular geometry

We have shown that lobular geometry is more difficult to assess in rodents and human due to the lack of clear boundaries. Lobular boundaries can be identified by the radial structure of the sinusoids. However, quantitative morphometric analysis of lobular architecture is challenging. Mice and rats are quite similar, making it difficult, even for experienced pathologists, to differentiate between the livers of the two species by morphological criteria. In contrast to pigs, the portal area of the terminal vessels lacks extracellular connective tissue and contains few histiocytes. Arteries in rodent livers are poorly visualized in contrast to those in human and porcine livers. As a result, human lobular geometry appears to be closer to that of rodents than to that of pigs. However, hepatic arteries are clearly visible due to the distinct appearance of the muscular vessel wall.

Furthermore, lobular geometry is a rather robust and stable parameter with little variation within the liver, i.e., between liver lobes, subjects, and more pronounced differences between species. The area and perimeter of the lobules increase with increasing species size, from mouse to rat, pig, and human.

Although the absolute number of liver lobules varies, the liver microarchitecture shows remarkable robustness across different species with only minor differences in the associated 2D geometric parameters. However, a high interlobular variability in geometric parameters was observed, as evident by the large standard deviations and interquartile ranges in all four species. One explanation could be the varying position, size, and 3-dimensional (3-D) shape of the lobule with respect to the 2-D sectioning plane. This finding has important implications for the computational modeling of the liver, allowing the reuse of lobular mathematical models of the liver across different species for simulation purposes with minor modifications (Ricken et al., 2015; Lambers et al., 2024).

The area and perimeter of the liver lobules were highly correlated (correlation coefficient *r* = 0.99), whereas compactness and area, respectively compactness and perimeter, were moderately and negatively correlated (*r* < 0.65). This finding suggests that the 3D structure of a hepatic lobule resembles possibly a more or less asymmetric ovaloid sphere. Here, more technical work is required to not only register a stack of stained sections to extract the 2D lobular shape but to go for a 3D reconstruction of entire hepatic lobules. This is needed to determine the 3D structure and packaging of the liver lobules in the context of the vascular tree. Reconstruction of the entire hepatic lobule, vascular tree, and sinusoidal network poses major technical challenges beyond our approach.

As part of the work, we have developed a robust method that allows non-rigid registration of a small stack of WSI sections, resulting in multiplexed protein images. While some spatial resolution is lost due to the registration, the results are sufficient to determine lobular geometries and zonation patterns of proteins, but not sufficient to resolve subcellular information. In particular, the presented workflow is easily applicable to immunofluorescence WSI images with multiple proteins, which would allow zonation patterns and lobular geometries to be determined with high spatial resolution.

The developed image processing workflow is a prerequisite for further imaging and modeling studies. For example, the established automatic registration and lobule segmentation without prior annotation is essential for extending the analysis to larger datasets as required for 3D reconstruction of the hepatic lobule and extraction of the vascular and sinusoidal network. Again, both are prerequisites for flow simulation using real lobular geometry, which in turn is required for further simulation of the perfusion-function relationship.

### 4.3 CYP expression

Drug metabolism studies have been performed for many years, both in humans and experimental animals involving different species. The underlying assumption is that the results obtained in animal studies reflect the clinical situation.

In our study, we selected a group of major CYP isoforms, CYP1A2, CYP2D6, CYP2E1, and CYP3A4, involved in the metabolism of many drug compounds. We focussed on these CYP enzymes since they are frequently used in drug metabolism studies involving CYP expression and *ex-vivo* activity levels but also in pharmacokinetic studies (Schenk et al., 2017; Albadry et al., 2022).

CYP3A is the predominant isoform in the CYP450 subfamily (Paine et al., 2006), metabolizing approximately 30%–50% of all clinically used drugs (Zhou, 2008; Tirona and Kim, 2017). The most widely used test drug specifically targeting CYP3A4 in pharmacokinetic studies is midazolam (Fuhr et al., 2007; Albadry et al., 2022). Evidence has been reported that expression levels are related to disease states, as reported for steatosis and carbon tetrachloride-induced liver injury (Hata et al., 2010; Schenk et al., 2017).

CYP2D6 is involved in the metabolism of approximately 20% of drugs (Vuppalanchi, 2018). Here, codeine, with its metabolites, is widely used as a test drug. Again, reduced expression levels are indicative of reduced drug elimination, as studied in CCL4-induced liver injury (Schenk et al., 2017).

CYP1A2 metabolizes 10%–15% of drugs, with caffeine being the most frequently used test compound (Fuhr et al., 2007; Vuppalanchi, 2018; Albadry et al., 2022). CYP1A2 activity is measured clinically to assess hepatic metabolic function. Here, the LiMAx assay is used, which is based on the metabolic turnover of 13C-labeled methacetin (Schurink et al., 2021).

CYP2E1 is the main enzyme in the CYP2E family. It has a crucial role in the metabolism of toxins such as CCl4 (Hammad et al., 2018), ethanol, and carcinogens and is involved in the metabolism of 5% of clinically important drugs such as acetaminophen (Shannon, 2007; Ghallab et al., 2019; Trousil et al., 2019; Hassan et al., 2024).

So far, little attention has been paid to the zonal expression of CYP proteins in different species, although it might be of high relevance to explain differences in drug metabolism and toxicity between species. We performed a systematic analysis of the zonal distribution and zonation patterns of four CYP450 enzymes (CYP1A2, CYP2D6, CYP2E1, and CYP3A4) in four species (mice, rats, pigs, and humans). Interspecies comparisons of the zonal distribution and expression of CYPs are very limited, with one study comparing the panlobular expression of CYP3A4 in the liver of adult minipigs with the reported pericentral to midzonal expression observed in humans (Van Peer et al., 2014).

It is highly likely that the extent and zonal distribution of CYP expression affects metabolic activity and may be critical for the interpretation of translational studies. We observed high similarities in zonal GS expression as well as in CYP2E1 expression between species. Our observation is in line with others (Martignoni et al., 2006), who reported a high degree of similarity in catalytic activity. The authors concluded that CYP2E1 activity did not show large differences between species, and extrapolation between species appeared to hold quite well. In contrast, the species-specific isoforms of CYP1A, CYP2C, CYP2D, and CYP3A show considerable interspecies differences in terms of expression pattern, as observed in our study, but also in catalytic activity, as reported by Martignoni et al. (2006) and Dalgaard (2015). These differences in the extent of expression and the catalytic activity could be the reason for the striking differences in hepatotoxicity.

Knowledge of the species-specific features of drug metabolism, such as differences and similarities in CYP patterns, will enable us to better predict therapeutic efficacy and toxicity according to species, and to generate safer and more effective therapeutic plans. It may also have a substantial impact on drug testing and preclinical drug development.

In summary, based on the observed similarities between animals and humans, mice appear to be the best choice for per-clinical experimental drug metabolism studies unless larger species are required, e.g., in studies investigating the effect of complex hepatobiliary surgery on drug metabolism. The selection of CYPs should be made according to the species being studied, with CYP2E1 being the preferred candidate when conducting cross-species studies in mice, rats, pigs, and humans. The selection of CYP1A2 seems reasonable when comparing rodents (mice and rats) with humans. If the study is limited to mice and humans, CYP3A4 is also of interest.

### 4.4 Minimal number of lobules

In clinical pathology, the evaluation of a minimum number of lobules is highly recommended to obtain a reliable pathological diagnosis. Misdiagnosis may occur due to limited sample size, especially when the recommended minimum number of portal fields is not obtained, the disease process is localized, and the interpreter lacks experience. The optimal length for a liver biopsy is reported to be 1–4 cm and a weight of 10–50 mg (Sherlock and Dooley, 2002). The majority of hepato-pathologists are satisfied with a biopsy specimen that includes at least six to eight portal triads, especially in cases of chronic liver disease where the extent of damage may vary among portal triads (Bravo et al., 2001). Agarwal et al. (2022) recently reported that a clinical liver biopsy should contain at least 10 complete portal fields to reliably diagnose allograft rejection (Agarwal et al., 2022). This number is based purely on pathological experience and not on detailed quantification of lobular geometry and quantification of staining patterns.

In this work, we determined the minimum number of lobules required to determine geometric parameters such as area and perimeter, protein zonation patterns, and percentage of stained area. Importantly, the minimum number of lobules required for a reliable quantitative analysis of lobular geometry varied with the given parameter and the acceptable margin of error for the estimate. The most robust parameter was compactness, requiring 2.2–3.8 lobules, whereas the area was less robust, requiring as many as 44.5–82.4 lobules for a confidence of 95% at a margin of error of 20%. The relatively large number of lobules required for a reliable estimate of the lobular geometry is a consequence of the large variability in the geometric parameters between different lobules from the same subject. This variability suggests that there is also some intrahepatic variability in terms of geometric parameters, resulting in slightly different numbers of lobules required for a given level of targeting accuracy in a given biopsy. In contrast, the mean values between individuals or even species are rather similar.

The calculation of the minimum number of lobules required to assess the zonated distribution also showed large differences between species. The main factor contributing to the large number of required lobules was the large coefficient of variation in the protein amount for the spatial locations, i.e., the more heterogeneous the zonation patterns are between different lobules on a sample, the higher the number of lobules needed for a reliable estimate. These differences in zonation patterns between different lobules could have important implications for toxicity or spatial drug metabolism.

When the same calculation was performed for CYP staining in terms of the normalized expression of a given stain, far fewer lobules were required to achieve the same level of confidence. Less than 10 lobules are required for rat and mouse, 10–20 lobules for pig, and 10–50 lobules for human. However, the number of lobules required depends on the protein of interest.

In this study, we only looked at normal livers in the four species. It is highly likely that the minimum number of lobules will increase in case of structural abnormalities. As expected, the higher the variability in the measurement of interest between different lobules, the more lobules are required for a reliable estimate of the parameter. Even simple parameters such as area, require a large number of lobules which can be obtained from WSI of a large liver sample, but not from a single liver core biopsy. Many parameters used in histopathological scoring systems have large spatial variability, which represents a challenge for reliable assessment.

Taken together, the minimum number of lobules is highly dependent on the species, the parameter, and probably the morphology of the liver. For many parameters, this number is much higher compared to the pathological standards for clinical diagnosis. Our results suggest that a quantitative analysis of clinical biopsies should be interpreted with caution. This may have implications for pathologic diagnosis, where the number of lobules determined here can never be achieved with a single biopsy.

### 4.5 Limitation of the study

In our species comparison, we looked only at adult subjects. Pigs (3 months) and rats (3 months) were young adults, whereas mice and humans were adults of moderate age (mice 8–9 m, Humans 45–59 years).

According to the literature, CYP mRNA and protein expression is stable in adults, see Supplementary Table 9. Therefore, it is reasonable to assume that zonal expression is also rather stable in adulthood. Kwak et al. (2015) performed a study in mice showing stable protein expression levels of CYP1A2 and 2E1 over an age range from 6 to 18 months. He reported that age-related changes begin to occur after 18 months, and become significant by 30 months of age (Kwak et al., 2015). These findings are valuable in understanding how the age of the subject may affect xenobiotic metabolism. Yun et al. (2010) found similar results in rats. He reported stable CYP reductase activity over an age range of 12–104 weeks. He also reported similar protein expression levels in adult rats aged 12 and 26 weeks but significantly lower levels in senescent rats at 104 weeks of age (Yun et al., 2010).

We also used samples from male and female pigs and humans. The collection of normal human samples was performed under strict conditions which made it difficult to extend our analysis to more subjects of the same sex. We did not observe any individual differences between pigs of different sexes. To further confirm this, we performed statistical analysis, which showed no statistically different results in the extent of zonated expression for GS and the 4 CYP enzymes between the group of three male versus three female pigs (see Supplementary Figures 1, 2).

Furthermore, it is important to note that our study was limited to a species comparison and did not take into account possible strain differences. Species differences, but also strain differences, could affect the level of expression and possibly other parameters of drug metabolism as described by (Kishida et al., 2008; McGill et al., 2012). This is best illustrated by comparing the results of Hassan and his group (2024) with ours. They observed a lower level of CYP2E1 expression in Wistar rats compared to C56Bl/6 mice (Hassan et al., 2024). In contrast, we observed exactly the same expression pattern in our C57Bl/6 mice, but to a higher extent in our Lewis rats. The 2008 study by Kishida and his group showed further significant differences in the mRNA expression of other CYP enzymes (CYP1A and 3A) between SD and Wistar rats. The researchers concluded that the observed differences in drug metabolism studies could be attributed to the rat strain used (Kishida et al., 2008). In conclusion, strain differences may have similar effects as species differences. Therefore, the search for the most appropriate animal model must take into account both the species, to select the appropriate size of animal to address the scientific question, and possibly the strain, to identify the one with the highest similarity to humans.

One could also ask whether the ethnicity of the human should be taken into account, as there may also be subtle differences when comparing samples from, for example, Caucasian versus African patients. The group of Guttman et al. (2019) examined the SNP polymorphism in CYP3A4 in different ethnicities using a large gene expression database (gnom AD browser) and observed substantial differences between ethnic groups (Guttman et al., 2019).

Furhermore, it is important to note that in addition to differences in CYP zoning, there are cross-species differences in the metabolism of certain drugs, for example, acetaminophen (APAP) is an analgesic and antipyretic drug that is metabolized by CYP3A4, 1A2, and 2E1 (Santoh et al., 2016). In mice, APAP is metabolized mainly by CYP2E1 and partially by CYP1A2 (Zaher et al., 1998; McGill and Jaeschke, 2013), whereas in humans, APAP is metabolized mainly by CYP3A4, followed by CYP2E1, CYP1A2, and CYP2D6, as suggested by (Laine et al., 2009). Overdoses of APAP produce the hepatotoxic metabolite N-acetyl-p-benzoquinone imine in mice and humans. In contrast to humans and mice, rats do not develop mitochondrial oxidative stress and, therefore, have minimal liver injury (Jaeschke et al., 2014). In our study, we found a similar CYP2E1 zonal expression in different species and animal strains used, with substantial differences in 1A2 and 3A4 zonal and gradient intensity expression. These differences in metabolism and CYP zonation may partly explain the failure to predict acetaminophen-induced hepatotoxicity in rats.

## 5 Conclusion

We presented an automated approach to assess lobular geometry and zonation patterns using WSI without the need for manual image annotation. This method allowed a detailed, systematic comparison of lobular structures and the zonal distribution and expression of key CYP enzymes (CYP1A2, CYP2D6, CYP2E1, and CYP3A4) and GS in different species (mouse, rat, pig, and human).

Based on the zonated expression of CYP enzymes, mice appear to be most similar to humans compared with the other species investigated. This finding should be taken into consideration when selecting a species for drug metabolism study. CYP selection should be based on species, with CYP2E1 and CYP2D6 being the most preferred when comparing four species. CYP1A2 could be considered as an additional CYP for rodent versus human comparisons, and CYP3A4 for mouse/human comparisons.

In conclusion, our image analysis pipeline together with suggestions for species and CYP selection, can serve to improve future cross-species and translational drug metabolism studies.

## Data Availability

The original contributions presented in the study are included in the article/Supplementary Material, further inquiries can be directed to the corresponding authors. Image analysis code is available from https://github.com/matthiaskoenig/zonation-image-analysis.
