# Supplementary material for: Cross-species variability in lobular geometry and cytochrome P450 hepatic zonation: insights into CYP1A2, CYP2D6, CYP2E1 and CYP3A4
Source: Front Pharmacol. 2024 May 16;15:1404938. doi: 10.3389/fphar.2024.1404938 (PMC11137285; doi:10.3389/fphar.2024.1404938)
Supplement: Supplementary file 1 [file DataSheet1.pdf]

Supplementary figures & tables

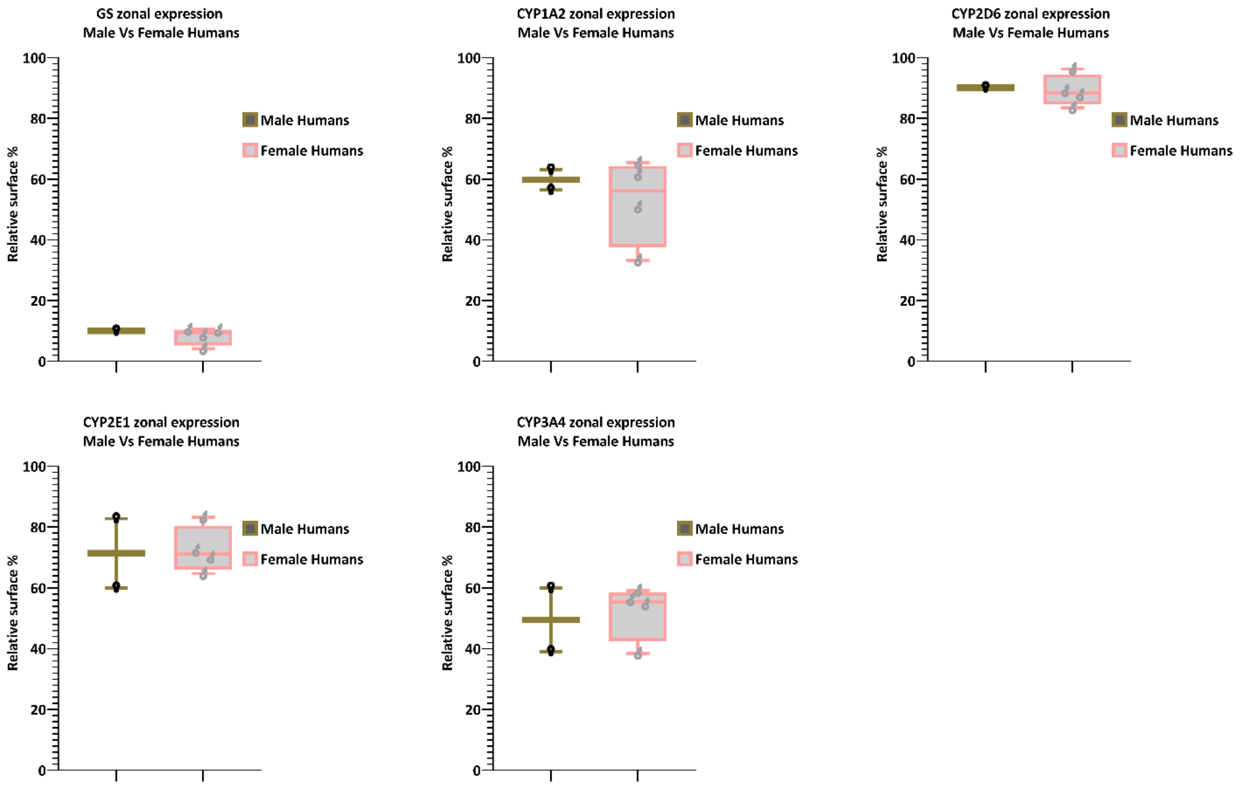

Supplementary Figure 1. Impact of gender on CYP expression in Human livers. Statistical analysis of GS and CYP zonal distribution in male vs female Humans (using unpaired nonparametric t-test (confidence level 95%, data in Box Plots figures shown as median with Interquartile range, performed using GraphPad Prism version 9.3.1(471) for Windows, a software developed by GraphPad Software, San Diego, California, USA, www.graphpad.com.)

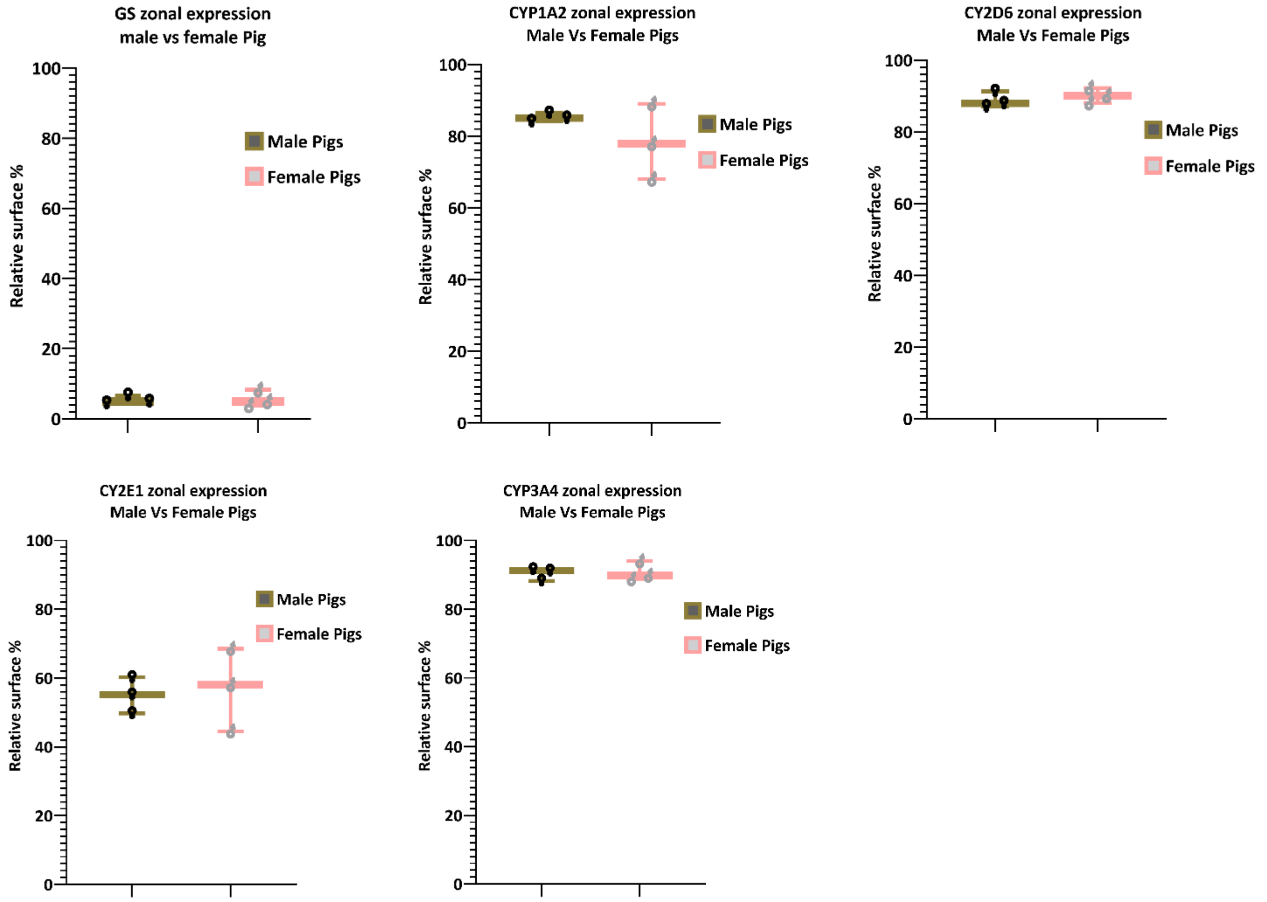

Supplementary Figure 2. Impact of gender on CYP expression in pigs livers. Statistical analysis of GS and CYP zonal distribution in male vs female pig (using unpaired nonparametric t-test (confidence level 95%, data in Box Plots figures shown as median with Interquartile range, performed using GraphPad Prism version 9.3.1(471) for Windows, a software developed by GraphPad Software, San Diego, California, USA, www.graphpad.com.)

This is a provisional file, not the final typeset article

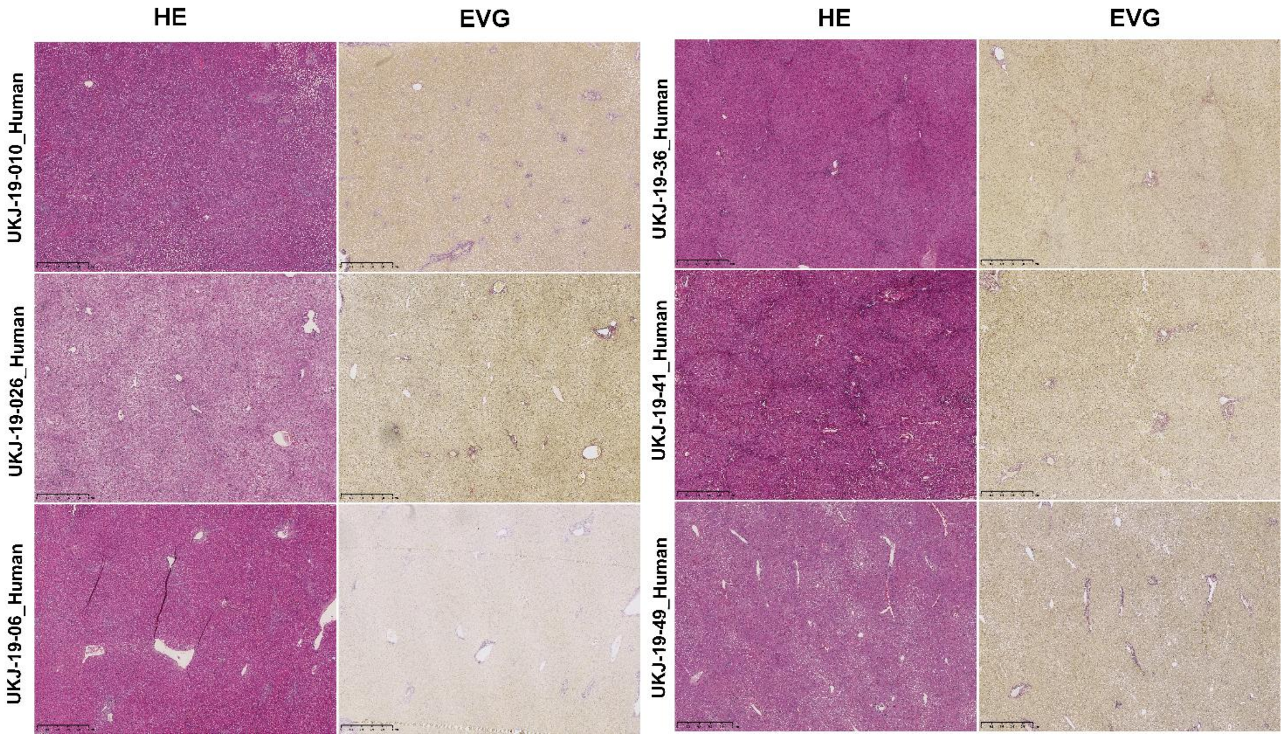

Supplementary Figure 3. HE and EVG staining of human liver tissue samples. (A) HE staining is used to assess morphology and steatosis. (B) EVG staining of human samples confirmed that they were fibrosis-free. Scale bar 1mm. Elastic fibers appear blue-black to black, Nuclei appear blue to black, collagen appears red, and other tissue elements appear yellow.

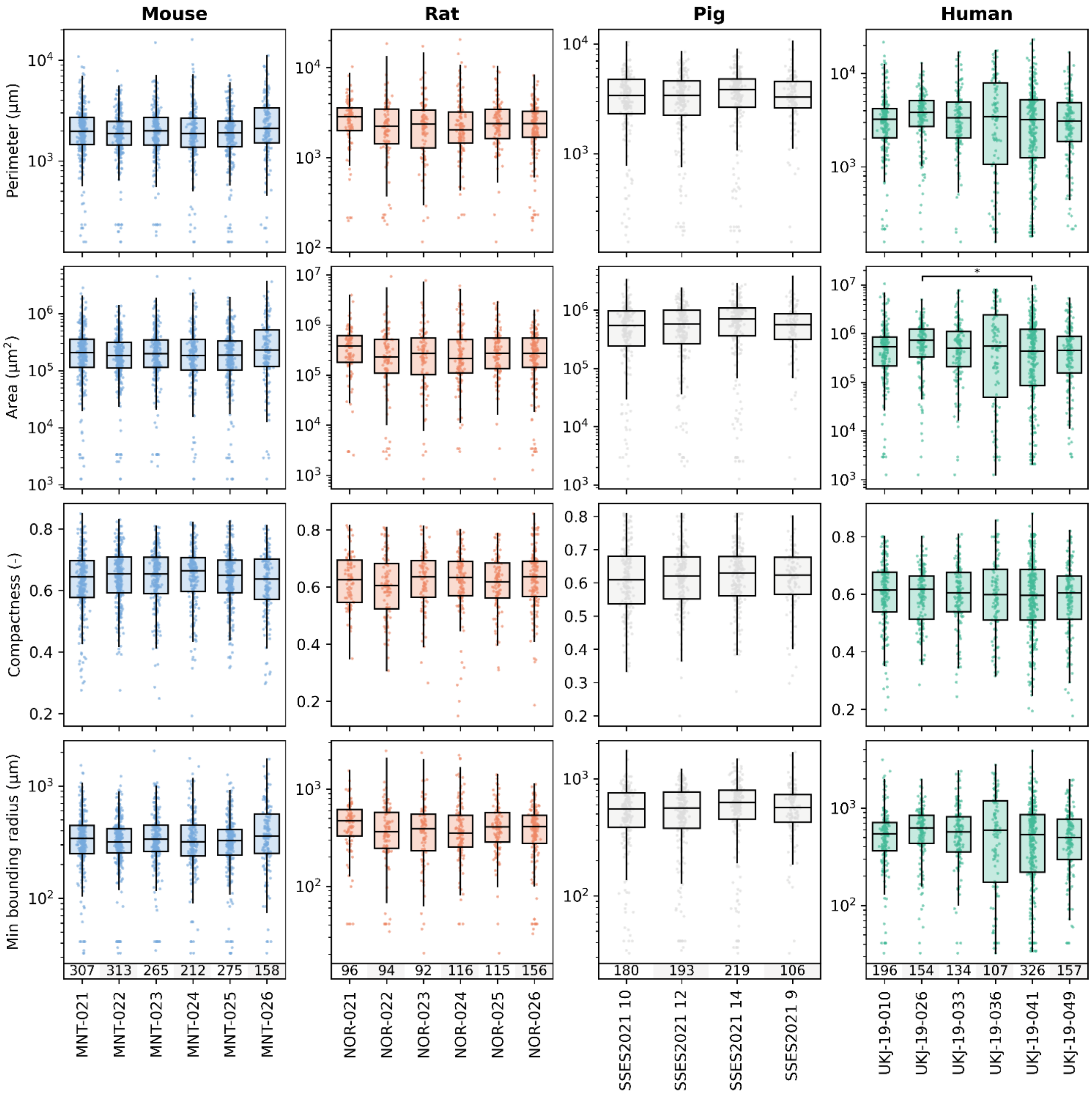

Supplementary Figure 4. Intra- and inter-individual variability of lobular geometric parameters in human, pig, rat and mouse. Boxes represent quantiles Q1 and Q3. Upper and lower whiskers extend to the last date less than Q3 + 1.5 * IQR and the first date greater than Q1 - 1.5 * IQR, respectively. IQR denotes interquartile range (Q3-Q1). Significance levels: * p<0.05, ** p<0.01, *** p<0.001, **** p<0.0001.

This is a provisional file, not the final typeset article

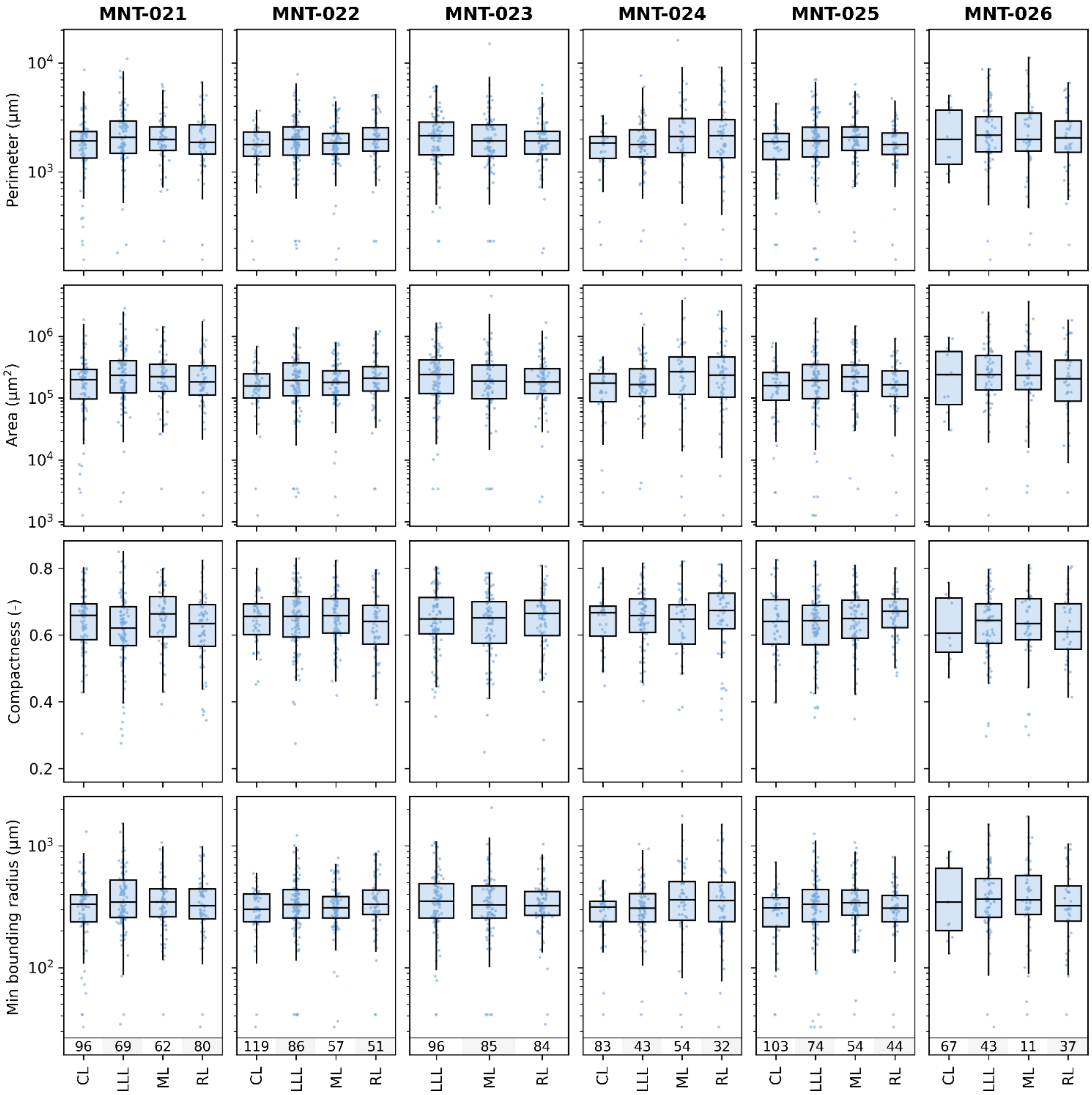

Supplementary Figure 5. Intra-lobe variability in lobular geometric parameters in mice. Boxes represent quantiles Q1 and Q3. Upper and lower whiskers extend to the last date less than Q3 + 1.5 * IQR and the first date greater than Q1 - 1.5 * IQR, respectively. IQR denotes interquartile range (Q3-Q1). Significance levels: * $p<0.05$, ** $p<0.01$, *** $p<0.001$, **** $p<0.0001$. CL: caudate lobe, LLL: left lateral lobe, ML: median lobe, right lobe; lCL in MNT-023 could not be evaluated due to lack of ROI registration.

Supplementary Table 1. Sample information for all species.

Subject	Species	Age	Strain	Gender	Fibrosis	Necro-inflammation	Steatosis
MNT-021	Mouse	8.9 months	Bl6/J	male	0	0	0
MNT-022	Mouse	8.9 months	Bl6/J	male	0	0	0
MNT-023	Mouse	8.9 months	Bl6/J	male	0	0	0
MNT-024	Mouse	8.9 months	Bl6/J	male	0	0	0
MNT-025	Mouse	9 months	Bl6/J	male	0	0	0
MNT-026	Mouse	9 months	Bl6/J	male	0	0	0
NOR-021	Rat	3.2 months	Lewis	male	0	0	0
NOR-022	Rat	3.2 months	Lewis	male	0	0	0
NOR-023	Rat	3.2 months	Lewis	male	0	0	0
NOR-024	Rat	3.2 months	Lewis	male	0	0	0
NOR-025	Rat	3.2 months	Lewis	male	0	0	0
NOR-026	Rat	3.2 months	Lewis	male	0	0	0
SSES2021/9	Pig	3 months	Prestice black-pied	female	0	0	0
SSES2021/10	Pig	3 months	Prestice black-pied	male	0	0	0
SSES2021/12	Pig	3 months	Prestice black-pied	male	0	0	0
SSES2021/14	Pig	3 months	Prestice black-pied	female	0	0	0
VS11	Pig	3 months	Prestice black-pied	male	0	0	0
VS12	Pig	3 months	Prestice black-pied	female	0	0	0
UKJ-19-026	human	45 years		female	0*	0	0
UKJ-19-036	human	54 years		male	0*	0	0
UKJ-19-033	human	55 years		female	0*	0	0
UKJ-19-049	human	55 years		female	0*	0	0
UKJ-19-041	human	59 years		female	0*	0	0
UKJ-19-010	human	56 years		male	0*	0	0

* for human samples HE and EvG staining was performed to confirm absence of fibrosis

Supplementary Table 2. Antibodies used for IHC-staining of GS and CYP enzymes for qualitative evaluation of expression pattern and signal intensity and quantitation of zonation (m for mouse, r for rat, p for pig, and h for human).

Antibody	Company	Order-Nr	Dilution/species	Detection systems
Anti-CYP2D6 (Rabbit polyclonal antibody)	Abcam, Germany	ab230690	1/3000 (m), 3000 (r), 1/2000 (p), 1/200 (H)	Rabbit-specific HRP/DAB Detection IHC Detection Kit - Micro-polymer (ab236469, Abcam, Germany)
Anti-CYP2E1 (Rabbit polyclonal antibody)	Sigma-Aldrich, Germany	HPA009128	1/400 (m), 1/300 (r), 1/800 (p), 1/800 (h)	
Anti-CYP3A4 (Rabbit polyclonal antibody)	Abcam, Germany	ab3572	1/2000 (m), 1/2000 (r), 1/1000 (p), 1/1500 (h)	
Anti-CYP1A2 (Mouse monoclonal antibody)	Abcam, Germany	ab22717	1/500 (m), 1/200 (r), 1/2000 (p), 1/2000 (h)	Dako Animal Research Kit Peroxidase for Mouse primary antibody, (K3954, Dako, Denmark)
Anti-GS (Mouse monoclonal antibody)	Merck, Germany	MAB302	1/1000 (m), 1/1000 (r), 1000 (p), 1/1000 (h)	

Supplementary Table 3. Overview of geometric parameters between species.

Species	Parameter	n	mean	sd	se	median	min	max	q1	q3	unit
mouse	perimeter	1530	2233	1397	57	1939	157	16155	1436	2636	µm
rat	perimeter	669	2734	2052	106	2345	116	20607	1534	3407	µm
pig	perimeter	698	3562	1823	135	3561	157	11024	2403	4695	µm
human	perimeter	1074	3966	3200	121	3365	157	23206	1841	5091	µm
mouse	area	1530	299281	360094	7651	192697	1269	4462695	110097	350281	$µm^2$

This is a provisional file, not the final typeset article

rat	area	669	467614	729369	18079	266095	846	9373817	121837	553765	µm^2
pig	area	698	718451	572536	27194	637528	1269	3788785	303006	1008009	µm^2
human	area	1074	966234	1357480	29484	530075	1269	10652257	157796	1174054	µm^2
mouse	compactness	1530	0.64	0.10	0.02	0.65	0.19	0.85	0.59	0.70	-
rat	compactness	669	0.62	0.11	0.02	0.63	0.15	0.86	0.56	0.69	-
pig	compactness	698	0.61	0.10	0.02	0.62	0.20	0.81	0.55	0.68	-
human	compactness	1074	0.59	0.12	0.02	0.61	0.18	0.88	0.52	0.68	-
mouse	minimum_bounding_radius	1530	375	216	10	330	33	2068	249	442	µm
rat	minimum_bounding_radius	669	451	299	17	401	21	2488	263	563	µm
pig	minimum_bounding_radius	698	583	285	22	581	33	1737	405	770	µm
human	minimum_bounding_radius	1074	637	473	19	559	33	3889	309	830	µm

Supplementary Table 4. Overview of required lobuli to determine geometric parameters in different species (with 95% confidence and a 20% margin of error).

species	perimeter	area (µm2)	compactness	minimum_bounding_radius (µm)
mouse	31.9 ± 11.5	82.0 ± 20.9	2.2 ± 1.1	27.8 ± 7.5
rat	34.4 ± 10.3	66.4 ± 9.2	2.7 ± 1.5	29.6 ± 8.9
pig	21.7 ± 7.3	44.5 ± 14.1	2.4 ± 0.8	20.0 ± 6.7
human	42.5 ± 29.4	82.4 ± 47.2	3.8 ± 2.9	37.6 ± 26.9

Supplementary Table 5. Overview of required lobuli to determine geometric parameters in different subjects.

species	subject	parameter	unit	mean	sd	n	n0.05	n0.1	n0.15	n0.2	n0.25	n0.3	n0.35
human	UKJ-19-010_Human	perimeter	µm	3741	2894	196	161.6	105.8	67.2	44.4	31.0	22.6	17.1
human	UKJ-19-026_Human	perimeter	µm	4000	2142	154	114.1	64.2	37.2	23.4	15.8	11.3	8.5
human	UKJ-19-033_Human	perimeter	µm	4017	3076	134	116.7	84.0	57.3	39.6	28.4	21.1	16.2
human	UKJ-19-036_Human	perimeter	µm	4940	4560	107	98.9	80.6	61.6	46.4	35.2	27.1	21.4
human	UKJ-19-041_Human	perimeter	µm	3873	3429	326	256.6	156.5	94.9	61.2	42.0	30.3	22.9
human	UKJ-19-049_Human	perimeter	µm	3701	2760	157	132.6	90.5	59.2	39.9	28.1	20.6	15.7
pig	SSES2021 10	perimeter	µm	3440	1811	180	126.5	66.9	37.5	23.2	15.6	11.1	8.3

pig	SSES2021 12	perimeter	µm	3418	1787	193	132.3	68.0	37.6	23.1	15.5	11.0	8.2
pig	SSES2021 14	perimeter	µm	3779	1862	219	138.0	65.4	34.9	21.1	14.0	9.9	7.4
pig	SSES2021 9	perimeter	µm	3584	1784	106	82.9	50.1	30.2	19.4	13.3	9.6	7.2
rat	NOR-021	perimeter	µm	2910	1612	96	79.8	52.9	33.9	22.5	15.8	11.5	8.7
rat	NOR-022	perimeter	µm	2636	2241	94	86.7	70.2	53.4	39.9	30.2	23.2	18.3
rat	NOR-023	perimeter	µm	2734	2266	92	84.6	68.2	51.6	38.4	28.9	22.2	17.5
rat	NOR-024	perimeter	µm	2890	2822	116	107.5	88.1	67.7	51.2	38.9	30.1	23.8
rat	NOR-025	perimeter	µm	2840	1808	115	97.1	66.1	43.2	29.1	20.5	15.0	11.4
rat	NOR-026	perimeter	µm	2493	1402	156	118.1	68.3	40.1	25.4	17.3	12.4	9.3
mouse	MNT-021	perimeter	µm	2273	1371	307	198.2	96.1	51.7	31.4	20.9	14.8	11.0
mouse	MNT-022	perimeter	µm	2072	1026	313	171.0	72.4	36.9	21.9	14.4	10.1	7.5
mouse	MNT-023	perimeter	µm	2243	1402	265	183.8	95.8	53.2	32.8	22.0	15.7	11.7
mouse	MNT-024	perimeter	µm	2276	1710	212	170.4	107.2	66.2	43.2	29.8	21.6	16.3
mouse	MNT-025	perimeter	µm	2089	1131	275	170.7	79.8	42.3	25.5	16.9	12.0	8.9
mouse	MNT-026	perimeter	µm	2645	1859	158	130.8	86.2	55.0	36.5	25.5	18.6	14.1
human	UKJ-19-010_Human	area	$µm^2$	837915	1264922	196	185.6	160.1	130.3	103.4	81.7	65.0	52.4
human	UKJ-19-026_Human	area	$µm^2$	878636	754435	154	135.6	99.8	69.3	48.5	35.0	26.1	20.1
human	UKJ-19-033_Human	area	$µm^2$	976494	1374596	134	128.4	113.9	96.0	78.6	63.8	51.9	42.5
human	UKJ-19-036_Human	area	$µm^2$	1656519	2217903	107	103.0	92.6	79.3	66.0	54.3	44.6	36.9
human	UKJ-19-041_Human	area	$µm^2$	942527	1350411	326	295.5	230.7	168.9	122.9	91.0	69.1	53.8
human	UKJ-19-049_Human	area	$µm^2$	782376	954124	157	146.9	123.2	97.0	74.8	57.8	45.2	36.0
pig	SSES2021 10	area	$µm^2$	664093	546665	180	153.5	106.4	70.4	47.8	33.8	24.9	19.0
pig	SSES2021 12	area	$µm^2$	679547	540088	193	161.0	107.5	69.2	46.2	32.3	23.7	18.0

This is a provisional file, not the final typeset article

pig	SSES2021 14	area	μm^2	808088	609002	219	175.1	109.3	67.2	43.7	30.1	21.8	16.5
pig	SSES2021 9	area	μm^2	696395	573430	106	96.2	75.3	55.3	40.3	29.9	22.7	17.7
rat	NOR-021	area	μm^2	506794	562745	96	91.4	79.8	65.9	53.0	42.4	34.0	27.6
rat	NOR-022	area	μm^2	460569	1007005	94	92.8	89.4	84.3	78.0	71.2	64.4	57.8
rat	NOR-023	area	μm^2	507442	925985	92	90.4	85.8	79.2	71.4	63.5	55.9	48.9
rat	NOR-024	area	μm^2	531143	903934	116	113.1	105.0	94.0	81.9	70.2	59.8	50.9
rat	NOR-025	area	μm^2	478036	566090	115	109.2	94.8	77.7	62.0	49.3	39.4	31.8
rat	NOR-026	area	μm^2	369337	333639	156	138.7	104.2	73.6	52.2	38.0	28.5	22.0
mouse	MNT-021	area	μm^2	301442	335951	307	264.5	186.8	125.4	85.9	61.1	45.2	34.6
mouse	MNT-022	area	μm^2	250421	222950	313	249.0	154.3	94.5	61.2	42.2	30.5	23.0
mouse	MNT-023	area	μm^2	299576	373616	265	238.6	183.6	132.6	95.5	70.3	53.1	41.2
mouse	MNT-024	area	μm^2	325437	468968	212	198.8	167.5	132.7	102.8	79.7	62.5	49.8
mouse	MNT-025	area	μm^2	262029	262374	275	233.3	160.4	105.5	71.3	50.3	37.0	28.2
mouse	MNT-026	area	μm^2	421125	515680	158	147.9	124.0	97.7	75.3	58.2	45.6	36.2
human	UKJ-19-010_Human	compactness	-	0.60	0.12	196	44.5	13.4	6.2	3.5	2.3	1.6	1.2
human	UKJ-19-026_Human	compactness	-	0.59	0.10	154	33.2	9.9	4.6	2.6	1.7	1.2	0.9
human	UKJ-19-033_Human	compactness	-	0.59	0.11	134	38.1	12.1	5.7	3.2	2.1	1.5	1.1
human	UKJ-19-036_Human	compactness	-	0.59	0.13	107	43.4	15.6	7.6	4.4	2.8	2.0	1.5
human	UKJ-19-041_Human	compactness	-	0.59	0.13	326	64.1	18.8	8.6	4.9	3.2	2.2	1.6
human	UKJ-19-049_Human	compactness	-	0.58	0.13	157	49.3	16.1	7.6	4.4	2.8	2.0	1.5
pig	SSES2021 10	compactness	-	0.61	0.10	180	35.5	10.4	4.8	2.7	1.8	1.2	0.9
pig	SSES2021 12	compactness	-	0.62	0.10	193	30.8	8.8	4.0	2.3	1.5	1.0	0.7
pig	SSES2021 14	compactness	-	0.62	0.09	219	30.8	8.6	3.9	2.2	1.4	1.0	0.7

pig	SSES2021 9	compactness	-	0.61	0.10	106	28.0	8.7	4.1	2.3	1.5	1.0	0.8
rat	NOR-021	compactness	-	0.62	0.09	96	25.1	7.8	3.6	2.1	1.3	0.9	0.7
rat	NOR-022	compactness	-	0.60	0.11	94	33.4	11.4	5.4	3.1	2.0	1.4	1.0
rat	NOR-023	compactness	-	0.62	0.10	92	28.4	9.2	4.3	2.5	1.6	1.1	0.8
rat	NOR-024	compactness	-	0.62	0.12	116	37.6	12.4	5.9	3.4	2.2	1.5	1.1
rat	NOR-025	compactness	-	0.61	0.10	115	29.0	8.9	4.2	2.4	1.5	1.1	0.8
rat	NOR-026	compactness	-	0.63	0.10	156	33.2	9.9	4.5	2.6	1.7	1.2	0.9
mouse	MNT-021	compactness	-	0.63	0.10	307	35.4	9.7	4.4	2.5	1.6	1.1	0.8
mouse	MNT-022	compactness	-	0.64	0.09	313	26.6	7.1	3.2	1.8	1.2	0.8	0.6
mouse	MNT-023	compactness	-	0.64	0.10	265	31.1	8.5	3.9	2.2	1.4	1.0	0.7
mouse	MNT-024	compactness	-	0.64	0.10	212	31.9	9.0	4.1	2.3	1.5	1.0	0.8
mouse	MNT-025	compactness	-	0.64	0.09	275	28.6	7.8	3.5	2.0	1.3	0.9	0.7
mouse	MNT-026	compactness	-	0.63	0.11	158	34.5	10.3	4.8	2.7	1.7	1.2	0.9
human	UKJ-19-010_Human	minimum_bounding_radius	µm	609	428	196	155.7	96.2	58.8	38.1	26.2	19.0	14.3
human	UKJ-19-026_Human	minimum_bounding_radius	µm	652	334	154	111.3	60.8	34.6	21.6	14.6	10.4	7.8
human	UKJ-19-033_Human	minimum_bounding_radius	µm	655	456	134	113.6	78.0	51.2	34.6	24.4	18.0	13.7
human	UKJ-19-036_Human	minimum_bounding_radius	µm	765	662	107	97.9	78.0	58.2	43.0	32.2	24.6	19.3
human	UKJ-19-041_Human	minimum_bounding_radius	µm	619	513	326	248.9	145.6	86.1	54.8	37.3	26.8	20.2
human	UKJ-19-049_Human	minimum_bounding_radius	µm	588	394	157	127.9	82.2	51.5	33.9	23.5	17.1	12.9
pig	SSES2021 10	minimum_bounding_radius	µm	557	280	180	123.0	63.1	34.8	21.4	14.3	10.2	7.6
pig	SSES2021 12	minimum_bounding_radius	µm	560	278	193	128.0	63.6	34.6	21.1	14.1	10.0	7.5
pig	SSES2021 14	minimum_bounding_radius	µm	620	293	219	133.7	61.7	32.5	19.6	12.9	9.1	6.8
pig	SSES2021 9	minimum_bounding_radius	µm	594	280	106	80.9	47.3	28.0	17.8	12.1	8.7	6.6

This is a provisional file, not the final typeset article

rat	NOR-021	minimum_bounding_radius	µm	486	255	96	78.2	50.3	31.6	20.7	14.4	10.5	7.9
rat	NOR-022	minimum_bounding_radius	µm	433	320	94	84.5	64.9	46.7	33.6	24.7	18.6	14.5
rat	NOR-023	minimum_bounding_radius	µm	446	329	92	82.9	63.9	46.2	33.3	24.5	18.5	14.4
rat	NOR-024	minimum_bounding_radius	µm	471	391	116	104.6	80.7	58.5	42.2	31.1	23.5	18.3
rat	NOR-025	minimum_bounding_radius	µm	467	270	115	94.0	60.7	38.2	25.1	17.5	12.7	9.6
rat	NOR-026	minimum_bounding_radius	µm	415	217	156	113.8	62.9	36.0	22.5	15.2	10.9	8.1
mouse	MNT-021	minimum_bounding_radius	µm	380	210	307	185.6	84.9	44.6	26.8	17.7	12.5	9.3
mouse	MNT-022	minimum_bounding_radius	µm	351	168	313	165.3	68.4	34.6	20.5	13.4	9.4	7.0
mouse	MNT-023	minimum_bounding_radius	µm	380	218	265	173.9	85.6	46.4	28.2	18.8	13.3	9.9
mouse	MNT-024	minimum_bounding_radius	µm	378	246	212	159.8	92.0	53.9	34.1	23.2	16.6	12.5
mouse	MNT-025	minimum_bounding_radius	µm	352	184	275	166.3	76.1	40.0	24.0	15.9	11.2	8.3
mouse	MNT-026	minimum_bounding_radius	µm	437	288	158	127.8	81.2	50.5	33.0	22.9	16.6	12.6

Supplementary Table 6. Overview of relative GS and CYP zonal expression [%].

Species	Subject	GS	CYP1A2	CYP2D6	CYP2E1	CYP3A4
Mouse	MNT-021	11.55	58.2	81.48	66.12	52.92
Mouse	MNT-022	12.32	57.47	89.12	67.25	51.58
Mouse	MNT-023	13.69	67.92	85.81	64.09	58.64
Mouse	MNT-024	10.65	52.95	68.41	60.53	66.21
Mouse	MNT-025	14.06	61.95	90.60	62.13	47.94
Mouse	MNT-026	7.05	61.28	74.43	47.45	63.52
Rat	NOR-021	6.23	29.25	93.71	59.84	47.09
Rat	NOR-022	6.79	33.61	78.80	59.81	33.69
Rat	NOR-023	8.12	41.21	97.55	72.60	72.25
Rat	NOR-024	6.81	30.99	95.00	69.50	53.21
Rat	NOR-025	6.73	24.99	95.54	57.81	84.50
Rat	NOR-026	9.64	34.92	91.78	72.91	62.79
Pig	SSES2021/10	4.94	86.44	87.97	60.18	91.56
Pig	SSES2021/12	4.47	85.07	87.08	55.13	88.24
Pig	SSES2021/14	4.99	68.01	92.23	44.55	88.82
Pig	SSES2021/9	8.30	89.02	90.06	68.51	93.99
Pig	VS11 3/8/21	6.71	84.12	91.28	49.70	91.18
Pig	VS12 3/8/21	3.86	77.90	88.06	58.01	89.83
Human	UKJ-19-026	4.12	33.31	89.15	70.02	54.69

Human	UKJ-19-036	10.04	63.14	90.19	82.80	59.94
Human	UKJ-19-033	10.49	61.43	83.52	72.36	38.45
Human	UKJ-19-049	8.58	50.80	96.31	83.18	59.20
Human	UKJ-19-041	10.21	65.39	87.76	64.70	56.14
Human	UKJ-19-010	NA	56.43	NA	60.03	39.11

Supplementary Table 7. Overview of statistical analysis of relative GS and CYP zonal expression (mean ± Sd) [%].

Species	GS	CYP1A2	CYP2D6	CYP2E1	CYP3A4
Mouse	11.55 ± 2.55	52.96 ± 5.05	81.64 ± 8.73	61.26 ± 7.2	56.8 ± 7.19
Rat	7.39 ± 1.27	32.5 ± 5.52	92.06 ± 6.78	65.41 ± 7	58.92 ± 18.2
Pig	5.55 ± 1.65	81.75 ± 7.68	89.45 ± 2.06	56.01 ± 8.36	90.6 ± 2.1
Human	8.69 ± 2.66	55.08 ± 11.88	89.39 ± 4.63	72.18 ± 7.4	51.26 ± 9.86

Supplementary Table 8. Overview of required lobuli to determine Relative GS and CYPs expression in different species (with 95% confidence and a 20% margin of error).

species	HE	GS	CYP1A2	CYP2D6	CYP2E1	CYP3A4
mouse	2.5 ± 1.5	20.1 ± 15.8	5.9 ± 2.0	2.0 ± 0.4	5.9 ± 1.6	4.4 ± 1.5
rat	8.1 ± 7.9	36.5 ± 9.5	8.9 ± 3.5	5.6 ± 1.1	7.1 ± 3.0	3.9 ± 1.3
pig	12.3 ± 8.1	28.9 ± 5.6	9.0 ± 4.7	7.3 ± 4.5	13.9 ± 3.5	4.0 ± 0.7
human	28.3 ± 20.0	42.1 ± 10.5	35.1 ± 16.4	16.8 ± 11.6	27.2 ± 22.1	30.6 ± 15.5

Supplementary Table 9. Impact of age on CYP-expression. A literature work up.

Author/year	Species	Age range	Zonation	Protein WB	Expression	Activity	Parameter
(Kwak et al., 2015)	C57Bl/6 mouse	2m, 6m, 18m, 30m	NA	↓ After 18month	↓ After 18month	↓ After 18month	CYP1A2,CYP1B1 CYP2B10, CYP2E1, CYP3A11
(Yun et al., 2010)	rat	3w, 12w, 26w, 104w	NA	Stable ↓ After 104 wks	NA	Stable	CYP1A2, CYP2B1, CYP2E1, CYP2C11, CYP3A2
(Hunt et al., 1992)	human		NA	NA	NA	Stable	CYP3A
(Yang et al., 2019)	Mixed gender SD Rat	1d, 7-14d, 21d, 28d 35d, 2m, 6m, 18m, 27m	NA	↓ After 27month	↓ After 27month	↓ After 27month	CYP1A1, CYP1A2, CYP2B1, CYP2B2, CYP2C6, CYP2C11, CYP2D2, CYP2E1, CYP3A1, CYP3A2, CYP4A1, CYP7A1, CYP7B1, CYP8B1, CYP27A1
(Wauthier et al., 2007)	Rat	9m and 24m	NA	↓ After 24month	↓ After 24month	↓ After 24month	CYP2E1 and 3A
(Wauthier et al., 2004)	Wistar Rat	3, 8, 11, 18m	NA	Stable till 11m	Stable till 11m	Stable till 11m	2E1 and 3A

This is a provisional file, not the final typeset article

References

Hunt, C.M., Westerkam, W.R., and Stave, G.M. (1992). Effect of age and gender on the activity of human hepatic CYP3A. *Biochem Pharmacol* 44(2)**,** 275-283. doi: 10.1016/0006-2952(92)90010-g.

Kwak, H.C., Kim, H.C., Oh, S.J., and Kim, S.K. (2015). Effects of age increase on hepatic expression and activity of cytochrome P450 in male C57BL/6 mice. *Arch Pharm Res* 38(5)**,** 857-864. doi: 10.1007/s12272-014-0452-z.

Wauthier, V., Verbeeck, R.K., and Buc Calderon, P. (2004). Age-related changes in the protein and mRNA levels of CYP2E1 and CYP3A isoforms as well as in their hepatic activities in Wistar rats. What role for oxidative stress? *Arch Toxicol* 78(3)**,** 131-138. doi: 10.1007/s00204-003-0526-z.

Wauthier, V., Verbeeck, R.K., and Calderon, P.B. (2007). The effect of ageing on cytochrome p450 enzymes: consequences for drug biotransformation in the elderly. *Curr Med Chem* 14(7)**,** 745-757. doi: 10.2174/092986707780090981.

Yang, J.Q., He, Y.Q., Zou, J.Y., Xu, L., Fan, F., and Ge, Z.L. (2019). Effect of Polygonum Multiflorum Thunb on liver fatty acid content in aging mice induced by D-galactose. *Lipids in Health and Disease* 18. doi: 10.1186/s12944-019-1055-y.

Yun, K.U., Oh, S.J., Oh, J.M., Kang, K.W., Myung, C.S., Song, G.Y., et al. (2010). Age-related changes in hepatic expression and activity of cytochrome P450 in male rats. *Arch Toxicol* 84(12)**,** 939-946. doi: 10.1007/s00204-010-0520-1.
